# Publisher Correction: Polymer-acid-metal quasi-ohmic contact for stable perovskite solar cells beyond a 20,000-hour extrapolated lifetime

**DOI:** 10.1038/s41467-024-46688-9

**Published:** 2024-03-14

**Authors:** Junsheng Luo, Bowen Liu, Haomiao Yin, Xin Zhou, Mingjian Wu, Hongyang Shi, Jiyun Zhang, Jack Elia, Kaicheng Zhang, Jianchang Wu, Zhiqiang Xie, Chao Liu, Junyu Yuan, Zhongquan Wan, Thomas Heumueller, Larry Lüer, Erdmann Spiecker, Ning Li, Chunyang Jia, Christoph J. Brabec, Yicheng Zhao

**Affiliations:** 1 National Key Laboratory of Electronic Films and Integrated Devices, School of Integrated Circuit Science and Engineering, University of Electronic Science and Technology of China, 611731 Chengdu, PR China; 2 Institute of Materials for Electronics and Energy Technology (i-MEET), Department of Materials Science, Friedrich-Alexander University Erlangen-Nürnberg, Martensstr. 7, 91058 Erlangen, Germany; 3 Shenzhen Institute for Advanced Study, University of Electronic Science and Technology of China, 518110 Shenzhen, PR China; 4 Institute of Micro- and Nanostructure Research & Center for Nanoanalysis and Electron Microscopy (CENEM), Department of Materials Science, FriedrichAlexander-Universität Erlangen-Nürnberg, Cauerstr. 3, D-91058 Erlangen, Germany; 5 Helmholtz-Institute Erlangen-Nürnberg (HI-ERN), Immerwahrstr. 2, 91058 Erlangen, Germany; 6 Institute of Polymer Optoelectronic Materials and Devices, State Key Laboratory of Luminescent Materials and Devices, South China University of Technology, 510640 Guangzhou, PR China

**Keywords:** Solar cells, Solar energy

Correction to: *Nature Communications* 10.1038/s41467-024-46145-7, published online 05 March 2024

In this article, Junsheng Luo and Bowen Liu should have been denoted as equally contributing authors. The original article has been corrected.

