# Supplementary material for: Polymer-acid-metal quasi-ohmic contact for stable perovskite solar cells beyond a 20,000-hour extrapolated lifetime
Source: Nat Commun. 2024 Mar 5;15:2002. doi: 10.1038/s41467-024-46145-7 (PMC10914746; doi:10.1038/s41467-024-46145-7)
Supplement: Supplementary file 1 — Supplementary Information [file 41467_2024_46145_MOESM1_ESM.pdf]

Supplementary Information

Polymer-acid-metal quasi-ohmic contact for stable perovskite solar cells beyond a 20,000-hour extrapolated lifetime

Junsheng Luo[1,2,3†], Bowen Liu[2†], Haomiao Yin[1], Xin Zhou[4], Mingjian Wu[4], Hongyang Shi[2], Jiyun Zhang[2,5], Jack Elia[2], Kaicheng Zhang[2], Jianchang Wu[2,5], Zhiqiang Xie[2], Chao Liu[2,5], Junyu Yuan[3], Zhongquan Wan[1,3*], Thomas Heumueller[2,5], Larry Lüer[2,5], Erdmann Spiecker[4], Ning Li[2,5,6], Chunyang Jia[1,3*], Christoph J. Brabec[2,5*] & Yicheng Zhao[1,5*]

[1]National Key Laboratory of Electronic Films and Integrated Devices, School of Integrated Circuit Science and Engineering, University of Electronic Science and Technology of China, 611731 Chengdu, P. R. China

[2]Institute of Materials for Electronics and Energy Technology (i-MEET), Department of Materials Science, Friedrich-Alexander University Erlangen-Nürnberg, Martensstr. 7, 91058 Erlangen, Germany

[3]Shenzhen Institute for Advanced Study, University of Electronic Science and Technology of China, 518110 Shenzhen, P. R. China

[4]Institute of Micro- and Nanostructure Research & Center for Nanoanalysis and Electron Microscopy (CENEM), Department of Materials Science, FriedrichAlexander-Universität Erlangen-Nürnberg, Cauerstr. 3, D-91058 Erlangen, Germany

[5]Helmholtz-Institute Erlangen-Nürnberg (HI-ERN), Immerwahrstr. 2, 91058 Erlangen, Germany

[6]Institute of Polymer Optoelectronic Materials and Devices, State Key Laboratory of Luminescent Materials and Devices, South China University of Technology, 510640 Guangzhou, P. R. China

[†]These authors contributed equally

*Correspondence to: zqwan@uestc.edu.cn; cyjia@uestc.edu.cn; christoph.brabec@fau.de; zhaoyicheng@uestc.edu.cn

This file includes:

Supplementary Figs. 1-32

Supplementary Tables 1-2

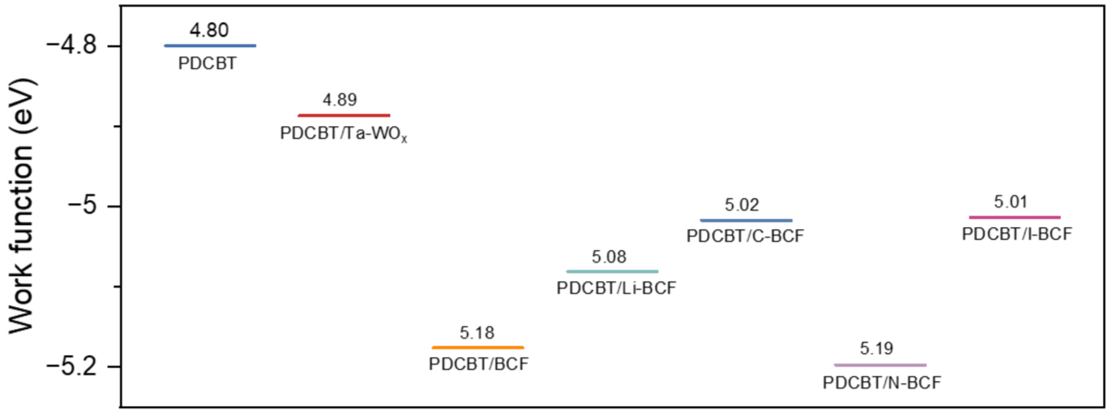

Supplementary Fig. 1 The *WF* values of PDCBT, PDCBT/Ta-WO_x, PDCBT/BCF, PDCBT/Li-BCF, PDCBT/C-BCF, PDCBT/N-BCF and PDCBT/I-BCF measured under dark in air.

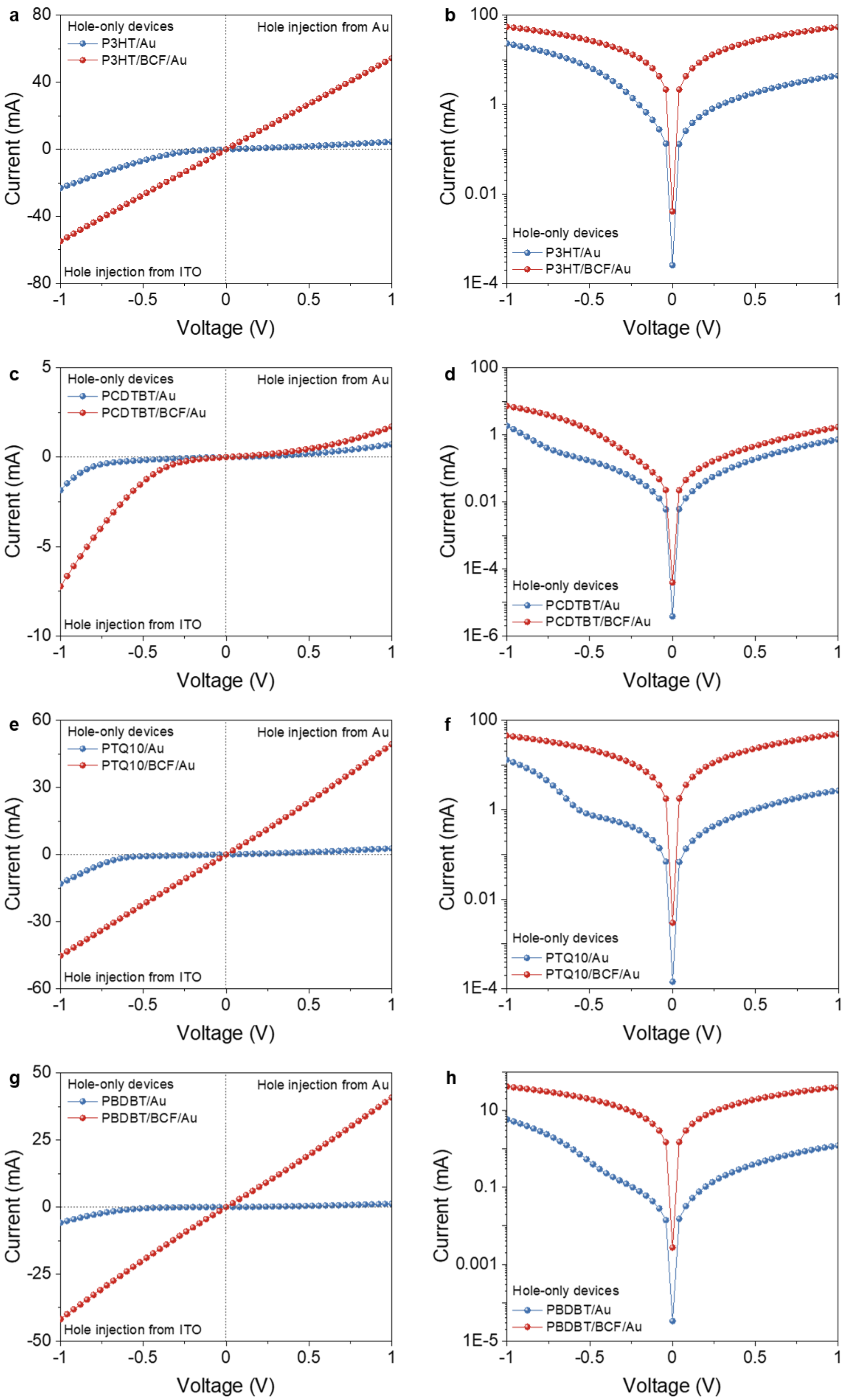
a
Hole-only devices
P3HT/Au
P3HT/BCF/Au
Hole injection from Au
Hole injection from ITO
Current (mA)
Voltage (V)
b
Hole-only devices
P3HT/Au
P3HT/BCF/Au
Current (mA)
Voltage (V)
c
Hole-only devices
PCDTBT/Au
PCDTBT/BCF/Au
Hole injection from Au
Hole injection from ITO
Current (mA)
Voltage (V)
d
Hole-only devices
PCDTBT/Au
PCDTBT/BCF/Au
Current (mA)
Voltage (V)
e
Hole-only devices
PTQ10/Au
PTQ10/BCF/Au
Hole injection from Au
Hole injection from ITO
Current (mA)
Voltage (V)
f
Hole-only devices
PTQ10/Au
PTQ10/BCF/Au
Current (mA)
Voltage (V)
g
Hole-only devices
PBDBT/Au
PBDBT/BCF/Au
Hole injection from Au
Hole injection from ITO
Current (mA)
Voltage (V)
h
Hole-only devices
PBDBT/Au
PBDBT/BCF/Au
Current (mA)
Voltage (V)

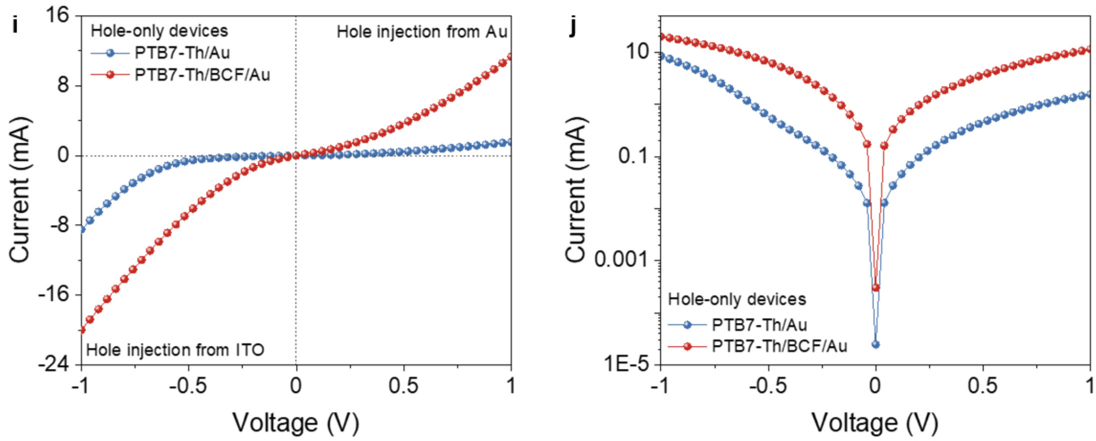

Supplementary Fig. 2 Current-voltage characteristics of ITO/PEDOT:PSS/*p*-type semicrystalline polymer/Au hole-only device with and without BCF interlayer between *p*-type semicrystalline polymer and Au. **a,b** P3HT as *p*-type semicrystalline polymer. **c,d** PCDTBT as *p*-type semicrystalline polymer. **e,f** PTQ10 as *p*-type semicrystalline polymer. **g,h** PBDBT as *p*-type semicrystalline polymer. **i,j** PTB7-Th as *p*-type semicrystalline polymer.

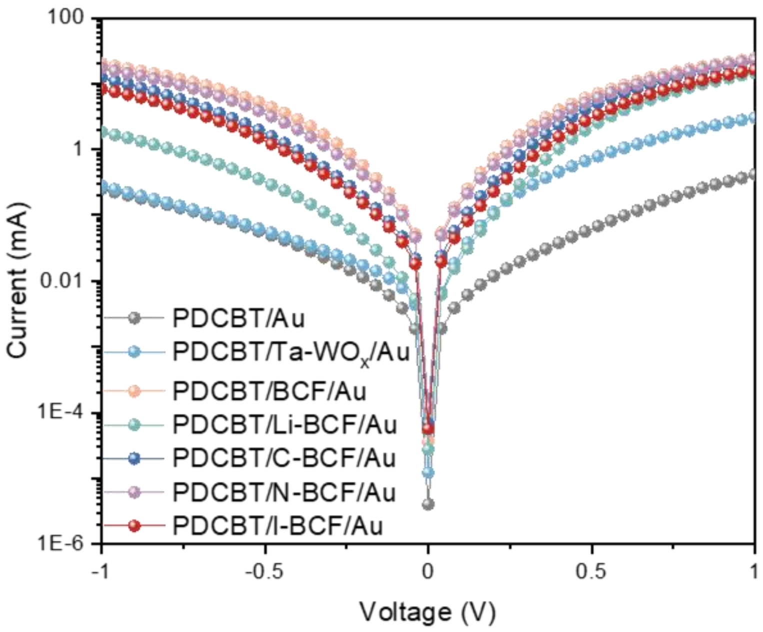

Supplementary Fig. 3 Vertical conductance of PDCBT with structure of ITO/PDCBT/Au and ITO/PDCBT/interlayer/Au.

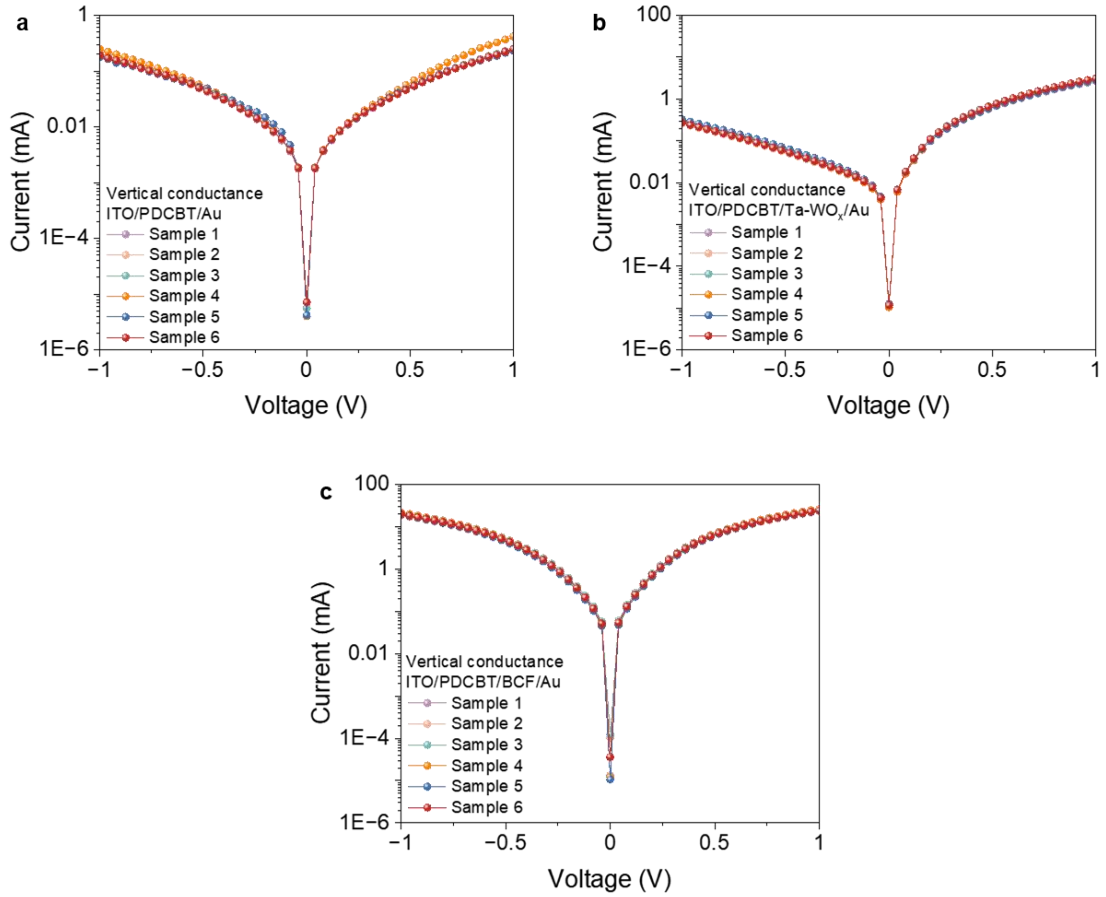

Supplementary Fig. 4 The reproducibility of vertical conductance. **a-c** The vertical conductance of devices based on architecture of (**a**) ITO/PDCBT/Au, (**b**) ITO/PDCBT/Ta-WO_x/Au and (**c**) ITO/PDCBT/BCF/Au.

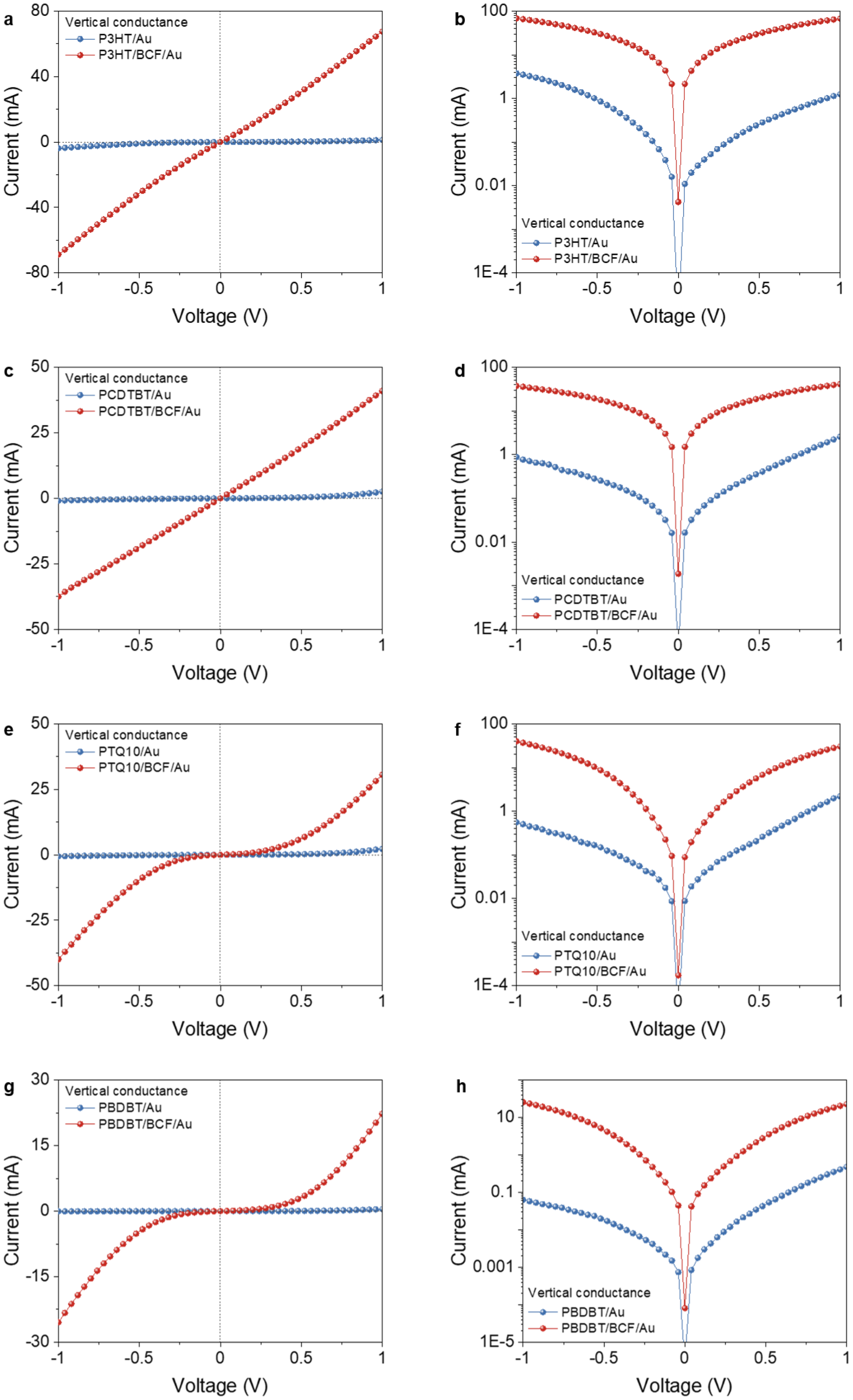
a
Vertical conductance
P3HT/Au
P3HT/BCF/Au
Current (mA)
Voltage (V)
b
Vertical conductance
P3HT/Au
P3HT/BCF/Au
Current (mA)
Voltage (V)
c
Vertical conductance
PCDTBT/Au
PCDTBT/BCF/Au
Current (mA)
Voltage (V)
d
Vertical conductance
PCDTBT/Au
PCDTBT/BCF/Au
Current (mA)
Voltage (V)
e
Vertical conductance
PTQ10/Au
PTQ10/BCF/Au
Current (mA)
Voltage (V)
f
Vertical conductance
PTQ10/Au
PTQ10/BCF/Au
Current (mA)
Voltage (V)
g
Vertical conductance
PBDBT/Au
PBDBT/BCF/Au
Current (mA)
Voltage (V)
h
Vertical conductance
PBDBT/Au
PBDBT/BCF/Au
Current (mA)
Voltage (V)

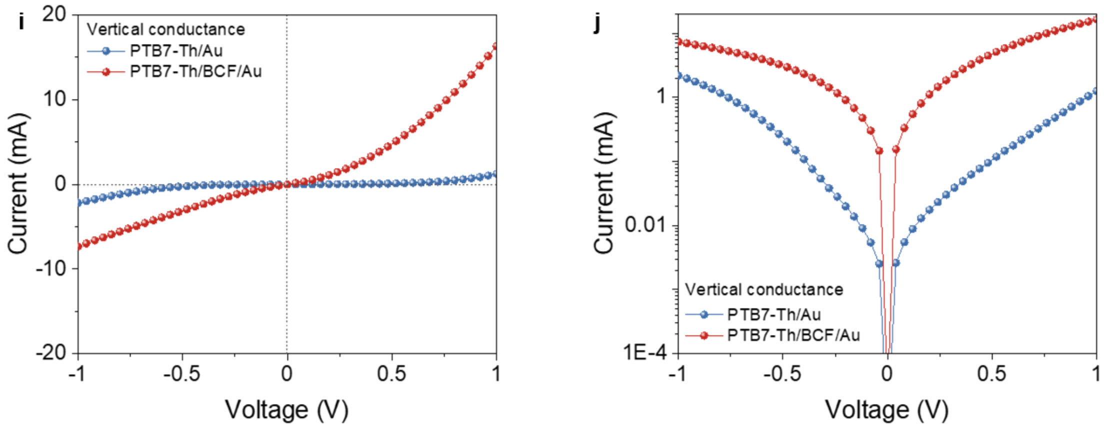

Supplementary Fig. 5 Vertical conductance with structure of ITO/*p*-type semicrystalline polymer/Au and ITO/*p*-type semicrystalline polymer/BCF/Au. **a,b** P3HT as *p*-type semicrystalline polymer. **c,d** PCDTBT as *p*-type semicrystalline polymer. **e,f** PTQ10 as *p*-type semicrystalline polymer. **g,h** PBDBT as *p*-type semicrystalline polymer. **i,j** PTB7-Th as *p*-type semicrystalline polymer.

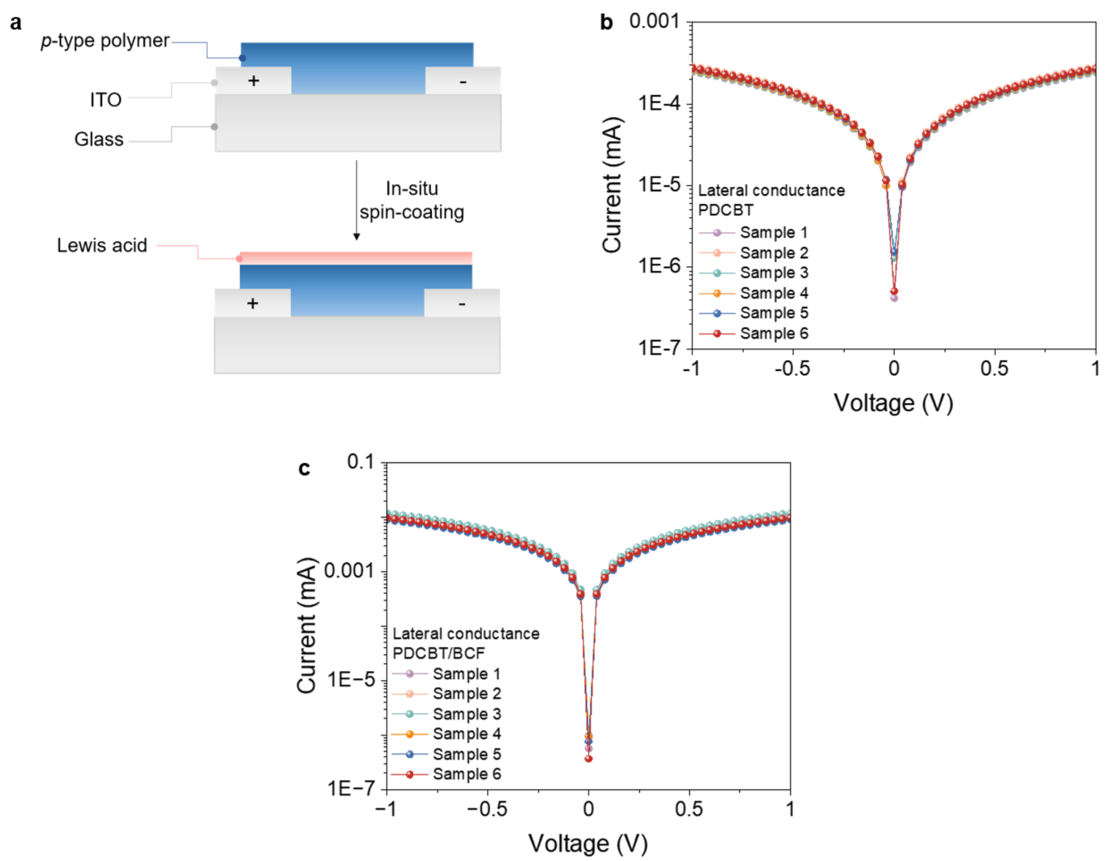

Supplementary Fig. 6 Lateral conductance. **a** Illustration of device architecture for measuring lateral conductance. **b-c** The reproducibility of lateral conductance of (**b**) PDCBT and (**c**) PDCBT/BCF samples.

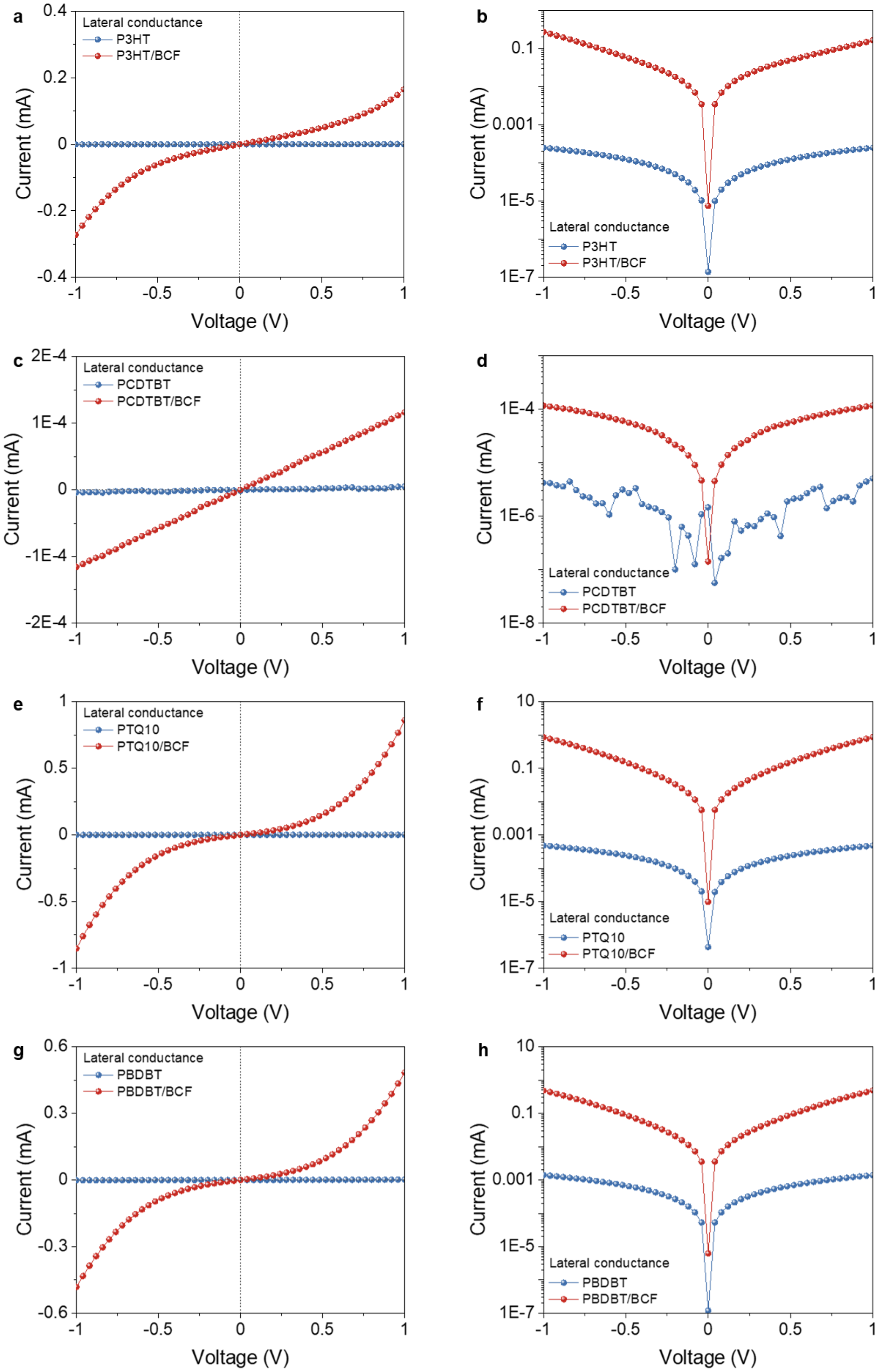
a
b
c
d
e
f
g
h
Lateral conductance
P3HT
P3HT/BCF
PCDTBT
PCDTBT/BCF
PTQ10
PTQ10/BCF
PBDBT
PBDBT/BCF
Current (mA)
Voltage (V)

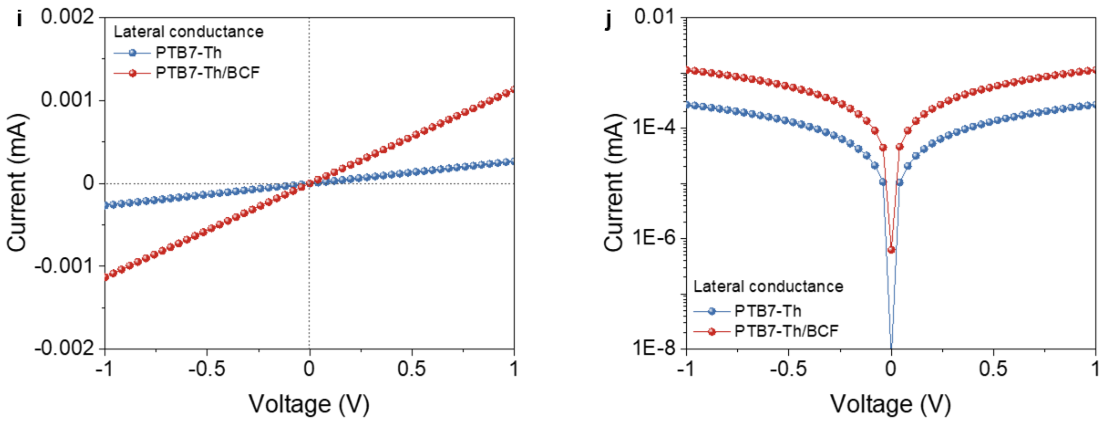

Supplementary Fig. 7 Current-voltage characteristics of lateral conductance with a structure of interdigitated ITO/*p*-type semicrystalline polymer/interdigitated ITO, and the change of lateral conductance of polymer after *in-situ* spin-coating BCF. **a,b** P3HT as *p*-type semicrystalline polymer. **c,d** PCDTBT as *p*-type semicrystalline polymer. **e,f** PTQ10 as *p*-type semicrystalline polymer. **g,h** PBDBT as *p*-type semicrystalline polymer. **i,j** PTB7-Th as *p*-type semicrystalline polymer.

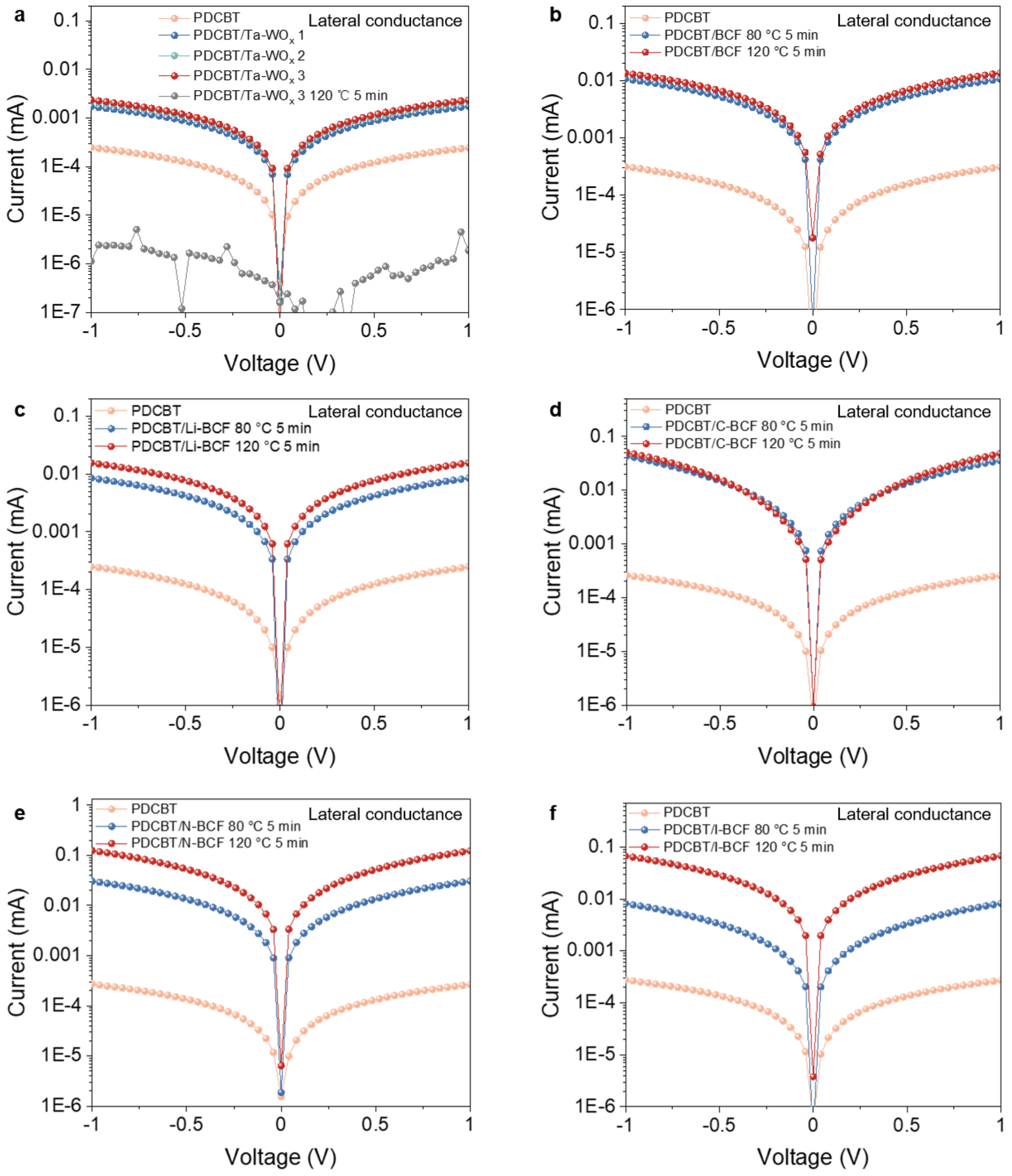

Supplementary Fig. 8 Lateral conductance. **a** The change of lateral conductance of PDCBT after sequentially *in-situ* spin-coating Ta-WO_x for three times without annealing and then annealing at 120 °C for 5 minutes. **b-f** The change of lateral conductance of PDCBT after sequentially *in-situ* spin-coating (**b**) BCF, (**c**) Li-BCF, (**d**) C-BCF, (**e**) N-BCF and (**f**) I-BCFand annealing at 80 °C for 5 minutes, 120 °C for 5 minutes, respectively.

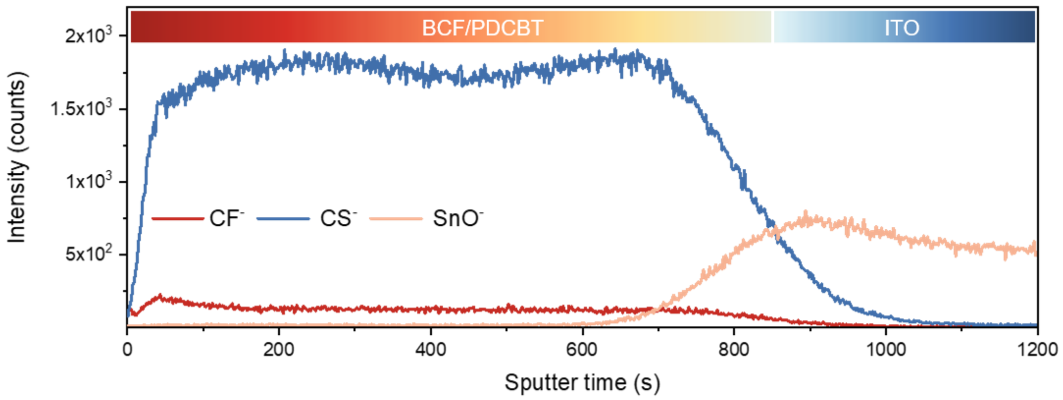

Supplementary Fig. 9 TOF-SIMS depth profile of the sample ITO/PDCBT/BCF.

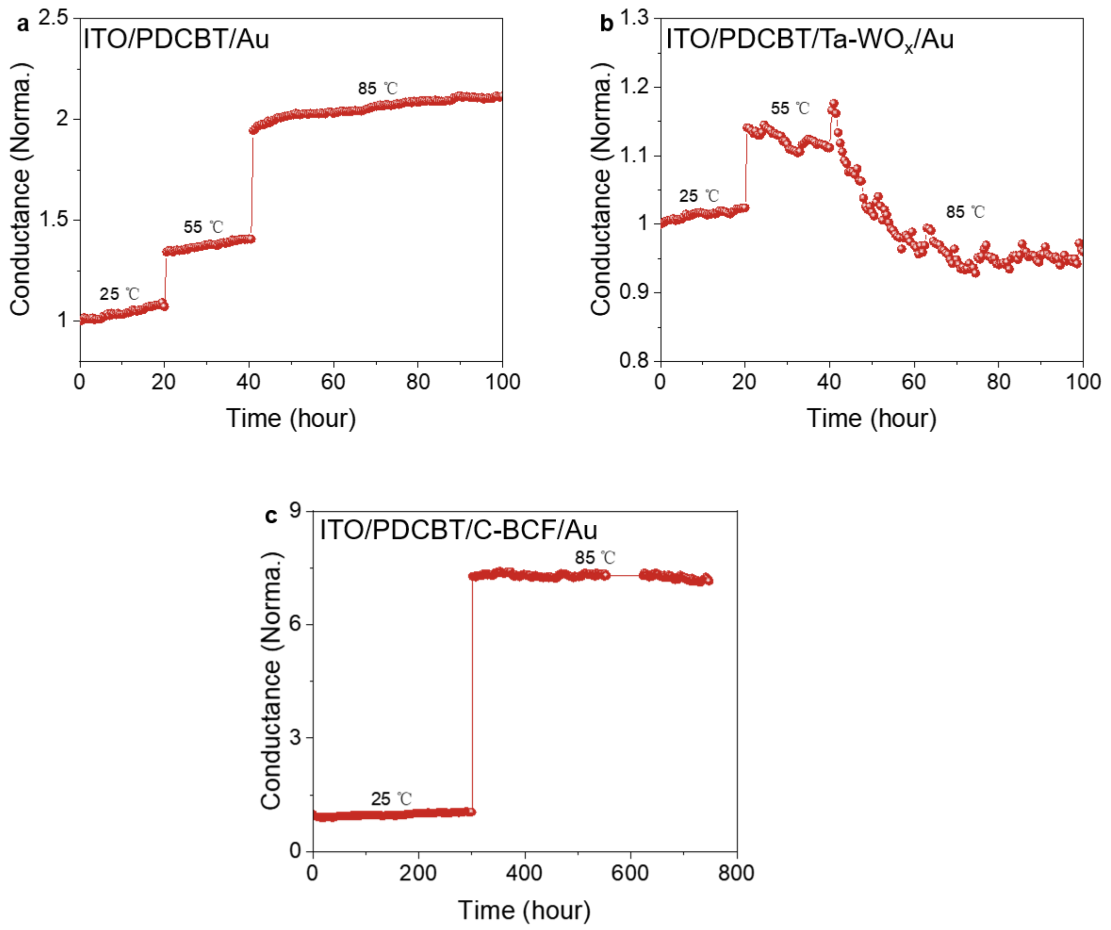

Supplementary Fig. 10 The long-term stability of vertical conductance at elevated temperatures in a N_2-filled chamber. **a-c** The conductance stability of (**a**) ITO/PDCBT/Au, (**b**) ITO/PDCBT/Ta-WO_x/Au and (**c**) ITO/PDCBT/C-BCF/Au.

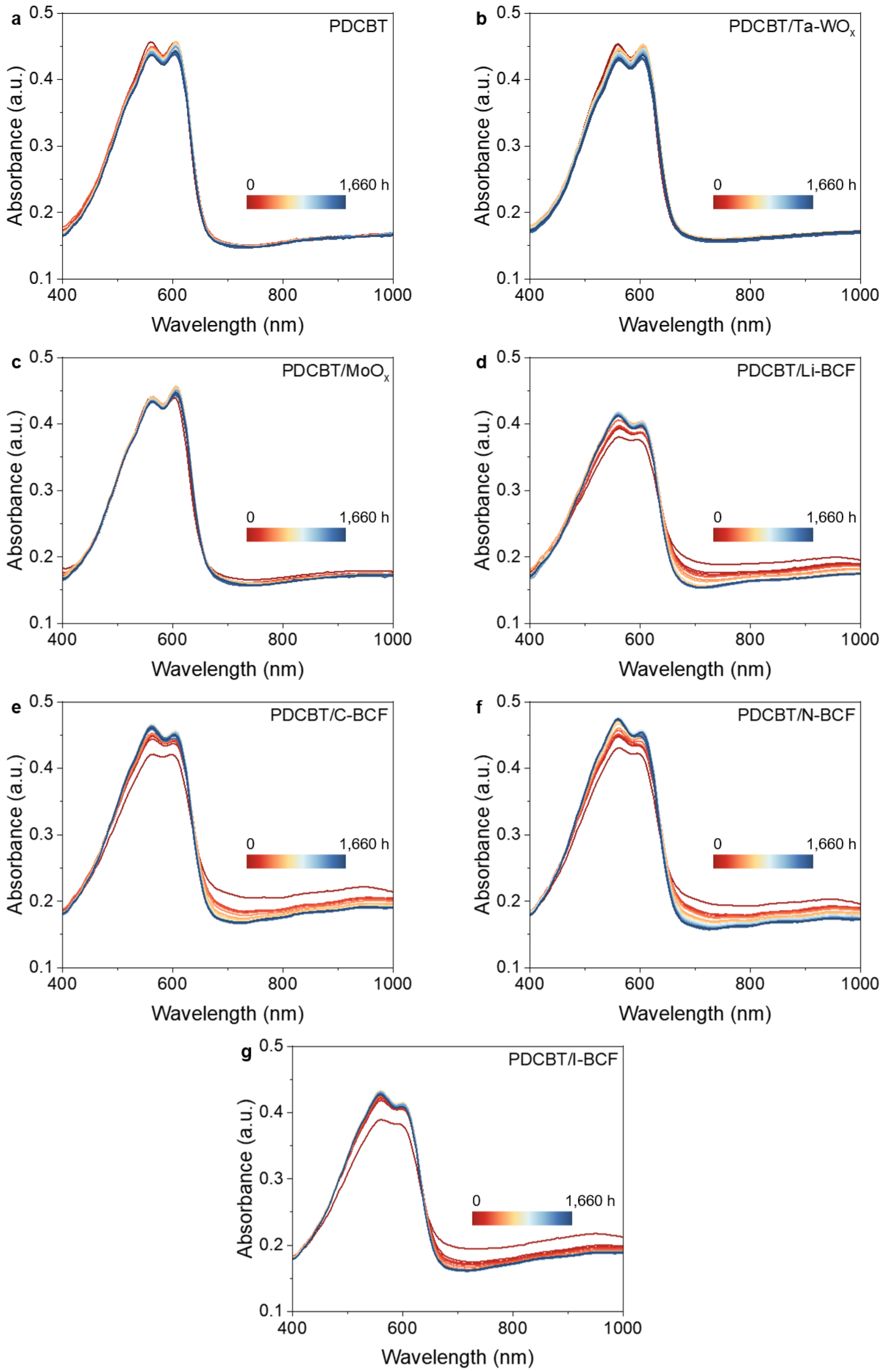

Supplementary Fig. 11 UV-vis absorption property of the encapsulated glass/function layer aging at 55 °C for 1,660 hours. **a-g** (**a**) PDCBT, (**b**) PDCBT/Ta-WOx, (**c**) PDCBT/MoOx, (**d**) PDCBT/Li-BCF, (**e**) PDCBT/C-BCF, (**f**) PDCBT/N-BCF and (**g**) PDCBT/I-BCF as function layer, respectively.

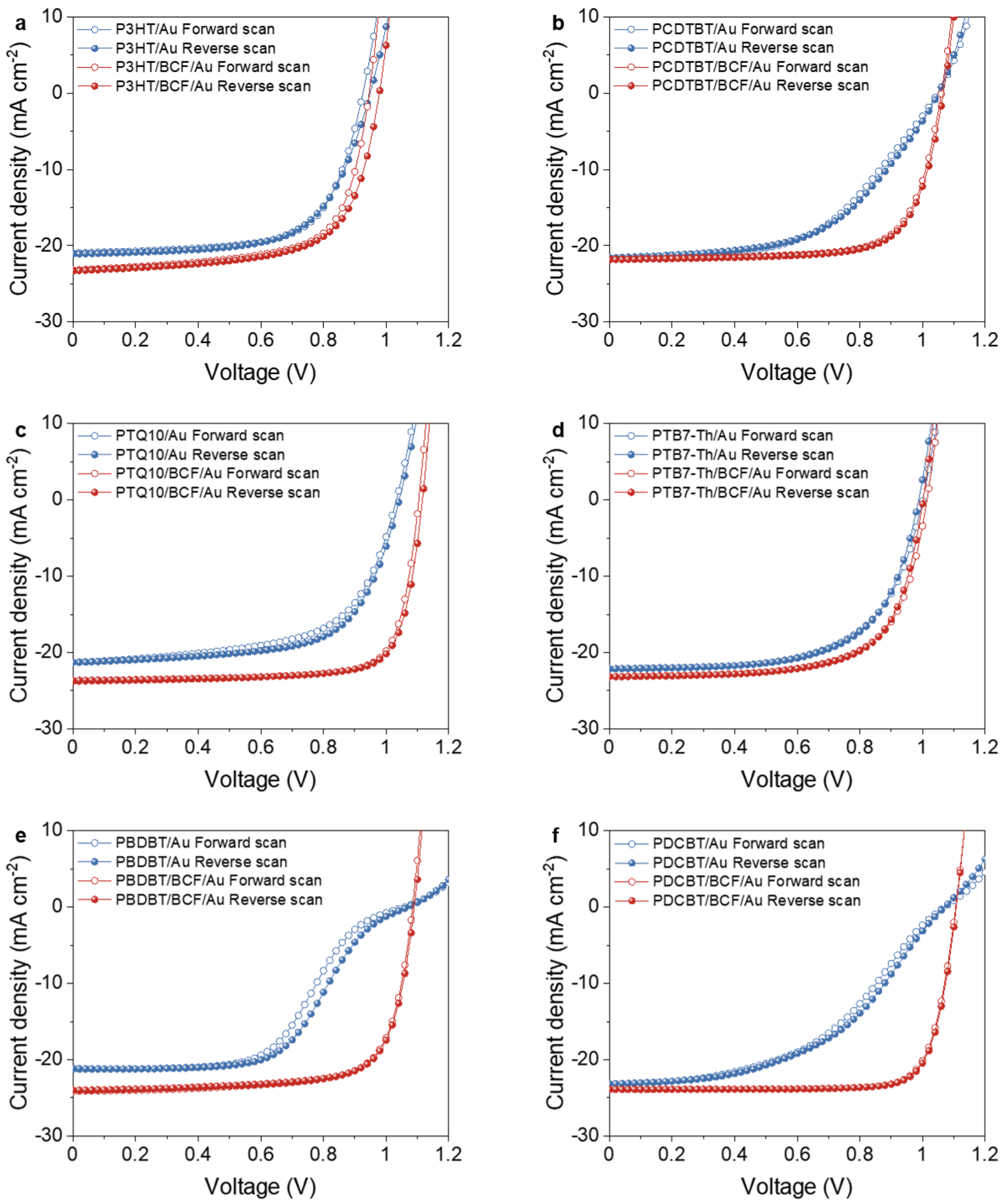

Supplementary Fig. 12 *J*-*V* curves of the champion PSCs with structure of ITO/SnO_2/PCBM/perovskite/*p*-type semicrystalline polymer/Au without and with BCF between *p*-type semicrystalline polymer and Au. **a-f** (**a**) P3HT, (**b**) PCDTBT, (**c**) PTQ10, (**d**) PTB7-Th, (**e**) PBDBT and (**f**) PDCBT as *p*-type semicrystalline polymer, respectively.

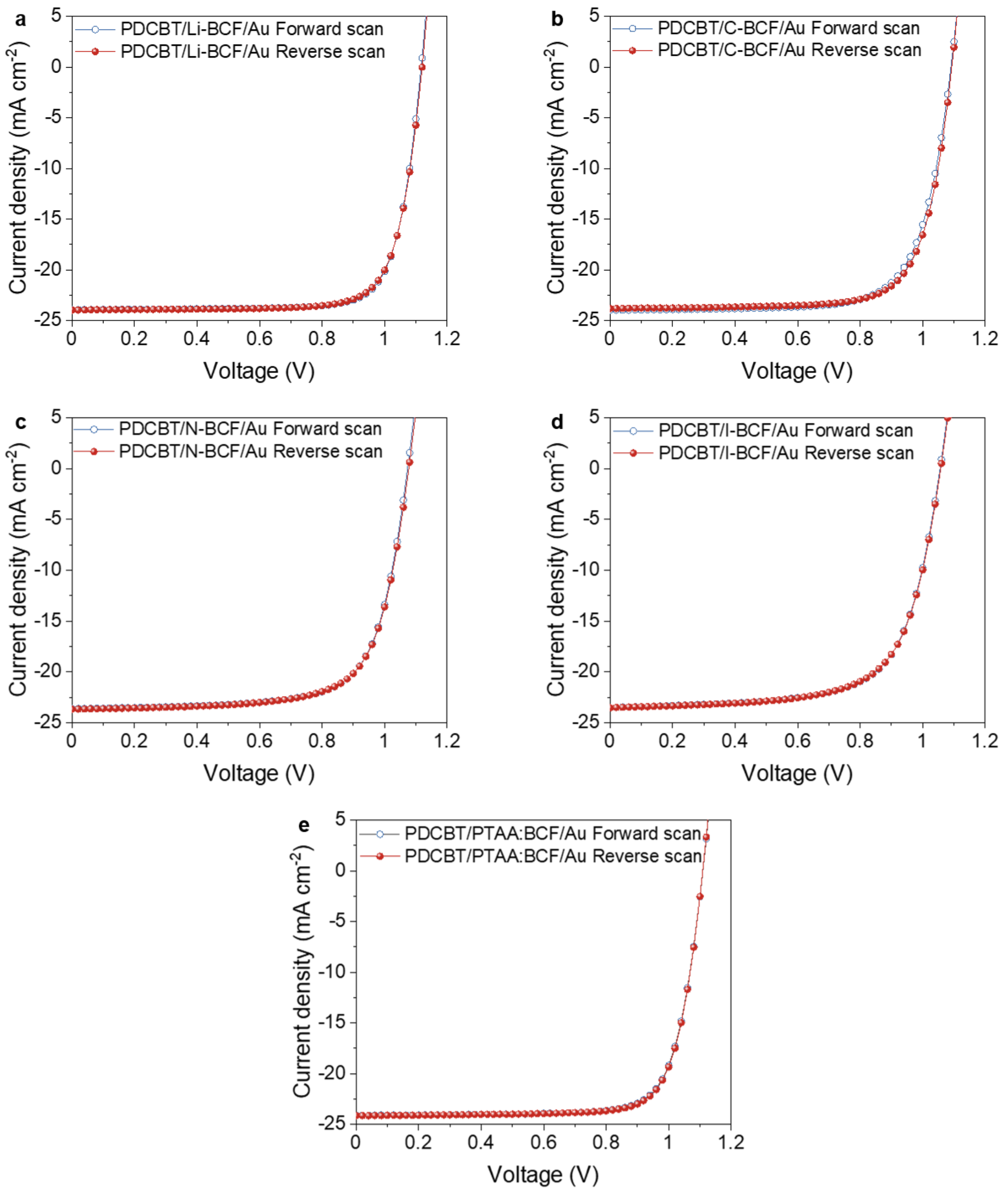

Supplementary Fig. 13 *J*-*V* curves of the champion PSCs with structure of ITO/SnO_2/PCBM/perovskite/PDCBT/quasi-ohmic contact layer/Au. **a-e** (**a**) Li-BCF, (**b**) C-BCF, (**c**) N-BCF, (**d**) I-BCF and (**e**) BCF doped PTAA as quasi-ohmic contact layer, respectively.

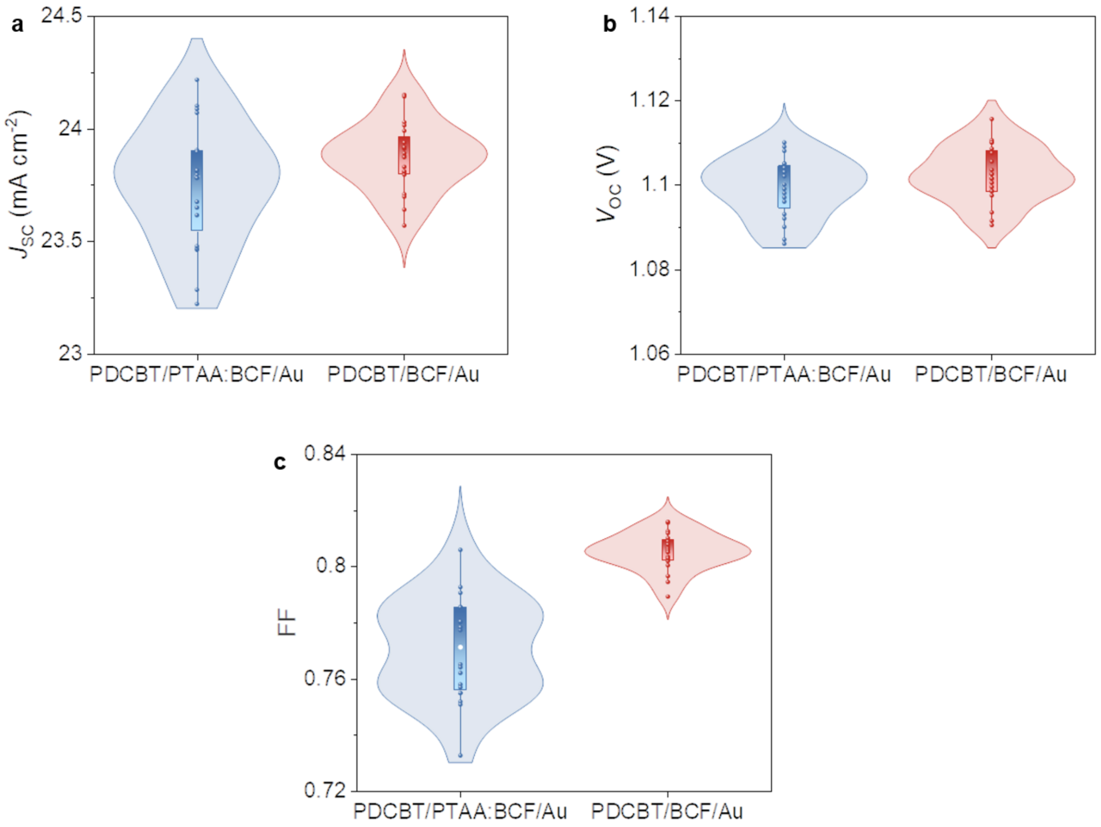

Supplementary Fig. 14 Statistical photovoltic performances of the PSCs based on ITO/SnO_2/PCBM/perovskite/PDCBT/PTAA:BCF or BCF/Au structure. **a-c** Statistical distribution of (**a**) J_{SC}, (**b**) V_{OC} and (**c**) FF.

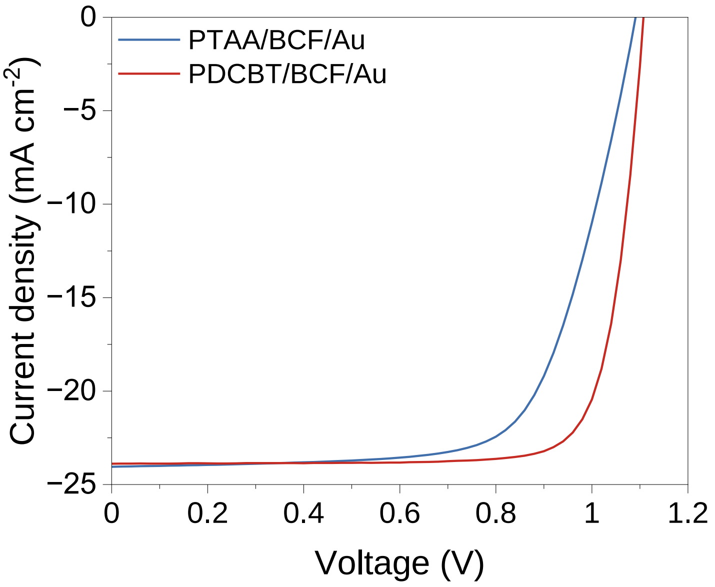

Supplementary Fig. 15 The *J-V* curves of PSCs based on ITO/SnO_2/PCBM/perovskite/HTL/Au structure using the BCF surface processing. Red curve: HTL = PDCBT/BCF, blue curve: HTL = PTAA/BCF.

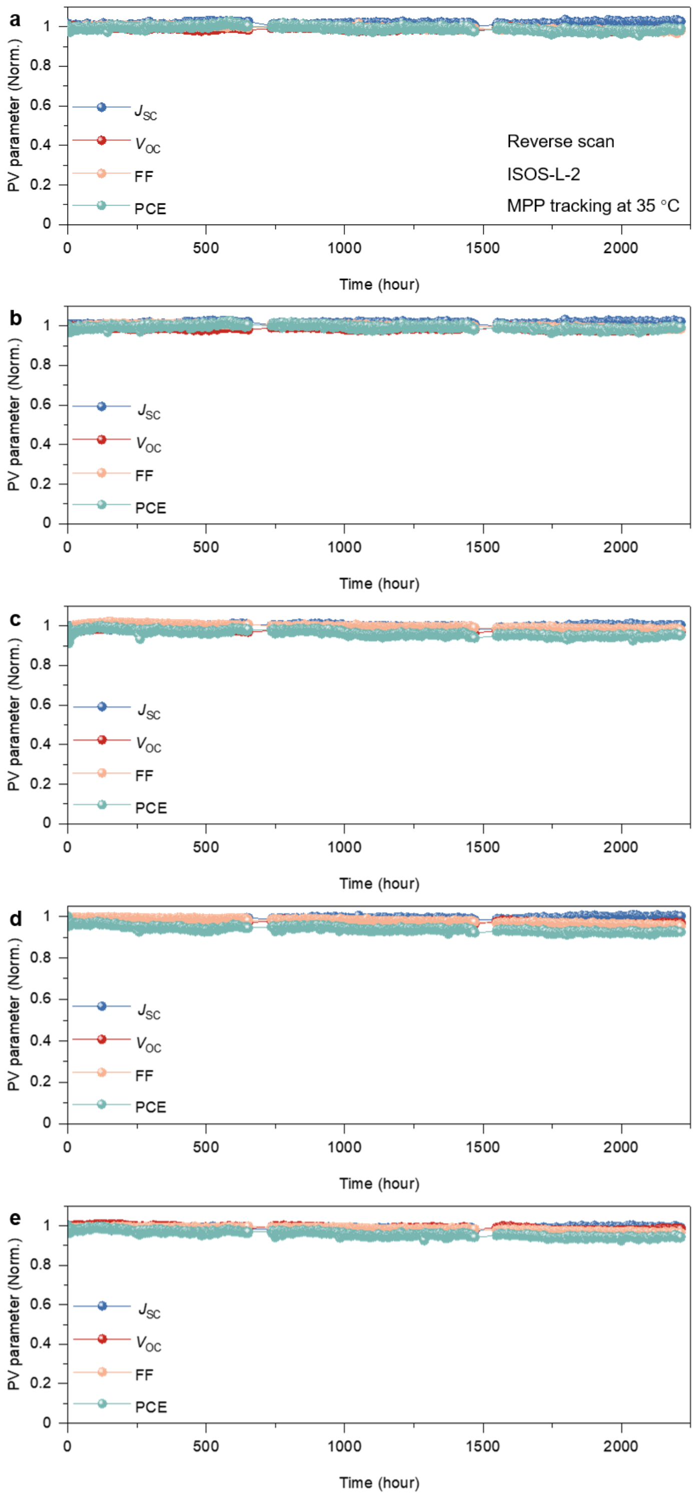

Supplementary Fig. 16 Normalized PV parameters of PSCs with structure of ITO/SnO_2/PCBM/perovskite/PDCBT/BCF/Au. **a-e** Normalized PV parameter of (**a**) device 1, (**b**) device 2, (**c**) device 3, (**d**) device 4 and (**e**) device 5 obtained from reverse *J-V* scans over time. The PSCs were aged at 35 °C in N_2-filled chamber under continuous light illumination (light intensity: 84 mW cm^{-2}).

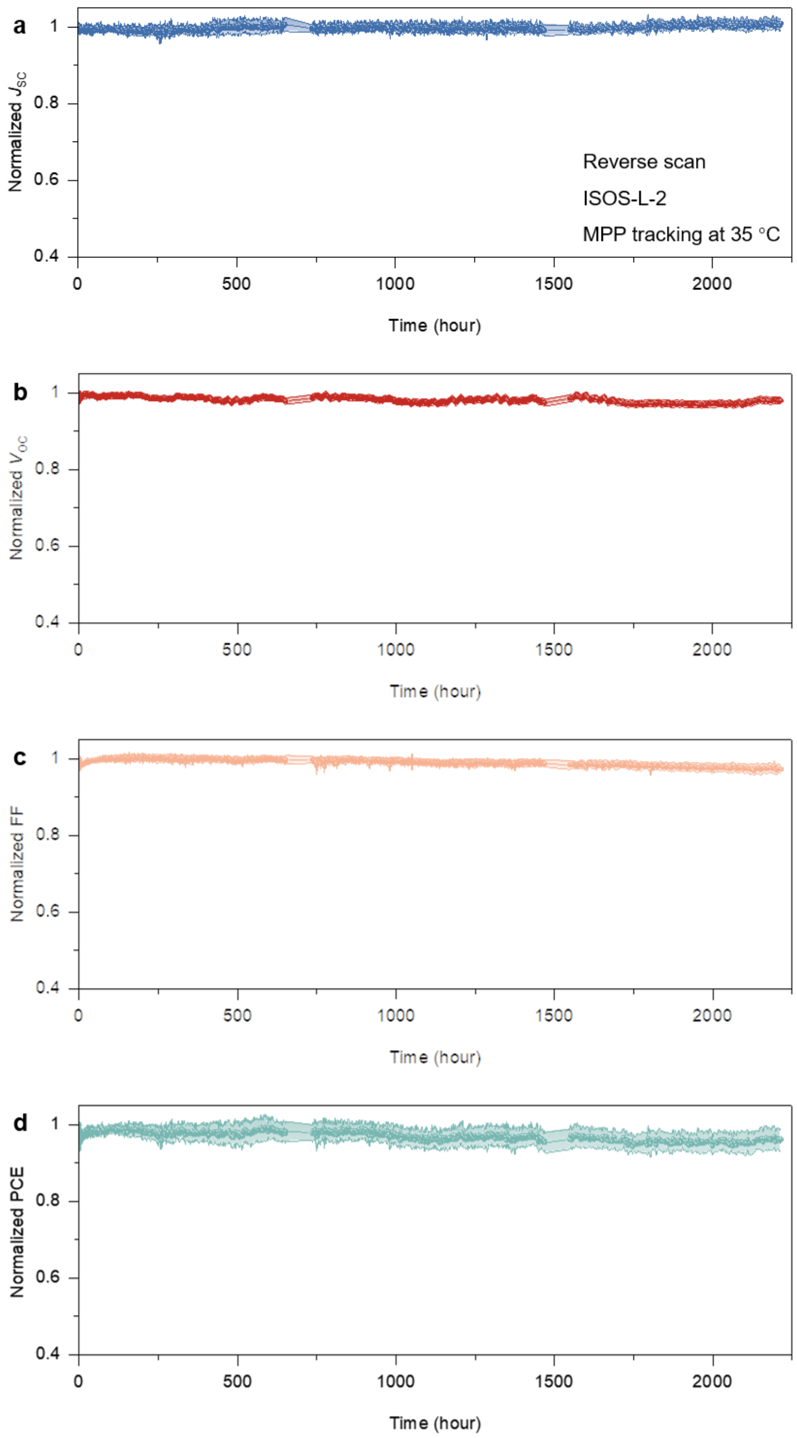

Supplementary Fig. 17 The statistic degradation behavior of PSCs with reverse *J-V* scan. **a-d** J_{SC} (**a**), V_{OC} (**b**), FF (**c**) and PCE (**d**) evolutions of PSCs based on ITO/SnO_2/PCBM/perovskite/PDCBT/BCF/Au architecture. The PSCs were aged at 35 °C in N_2-filled chamber under continuous light illumination (light intensity: 84 mW cm^{-2}). Error bars represent the standard deviations from the statistic results of 5 individual devices.

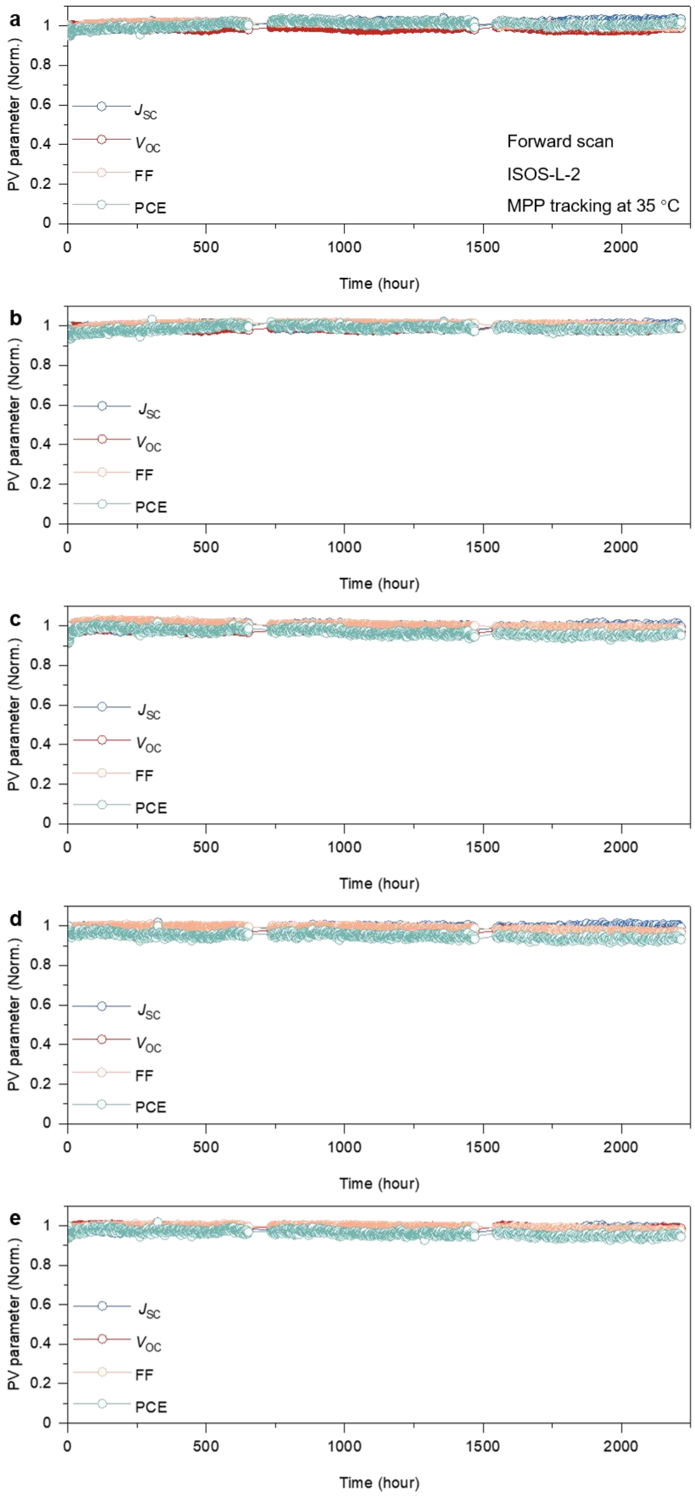

Supplementary Fig. 18 Normalized PV parameters of PSCs with structure of ITO/SnO_2/PCBM/perovskite/PDCBT/BCF/Au. **a-e** Normalized PV parameter of (**a**) device 1, (**b**) device 2, (**c**) device 3, (**d**) device 4 and (**e**) device 5 obtained from forward *J-V* scans over time. The PSCs were aged at 35 °C in N_2-filled chamber under continuous light illumination (light intensity: 84 mW cm^{-2}).

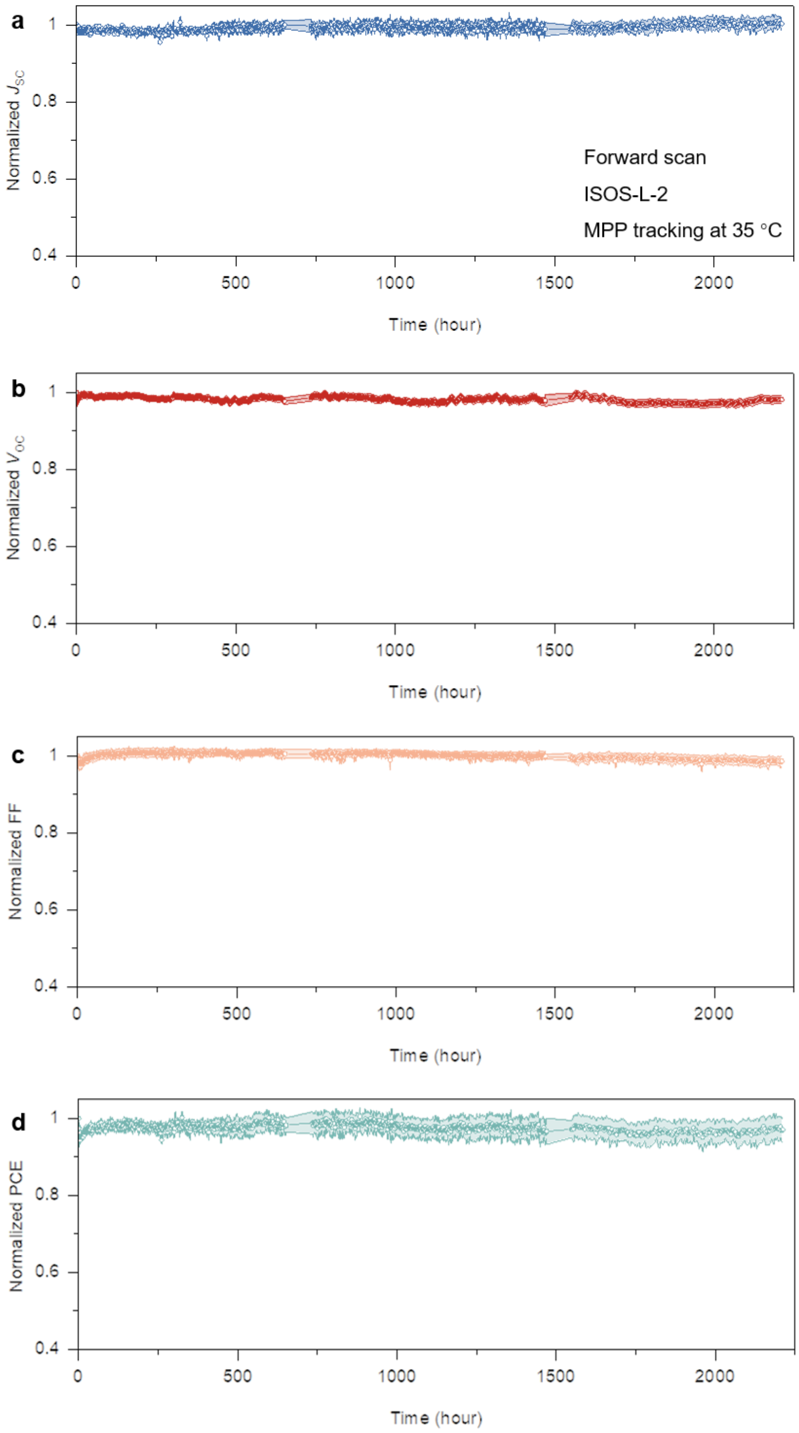

Supplementary Fig. 19 The statistic degradation behavior of PSCs with forward *J-V* scan. **a-d** J_{SC} (**a**), V_{OC} (**b**), FF (**c**) and PCE (**d**) evolutions of PSCs based on ITO/SnO_2/PCBM/perovskite/PDCBT/BCF/Au architecture. The PSCs were aged at 35 °C in N_2-filled chamber under continuous light illumination (light intensity: 84 mW cm^{-2}). Error bars represent the standard deviations from the statistic results of 5 individual devices.

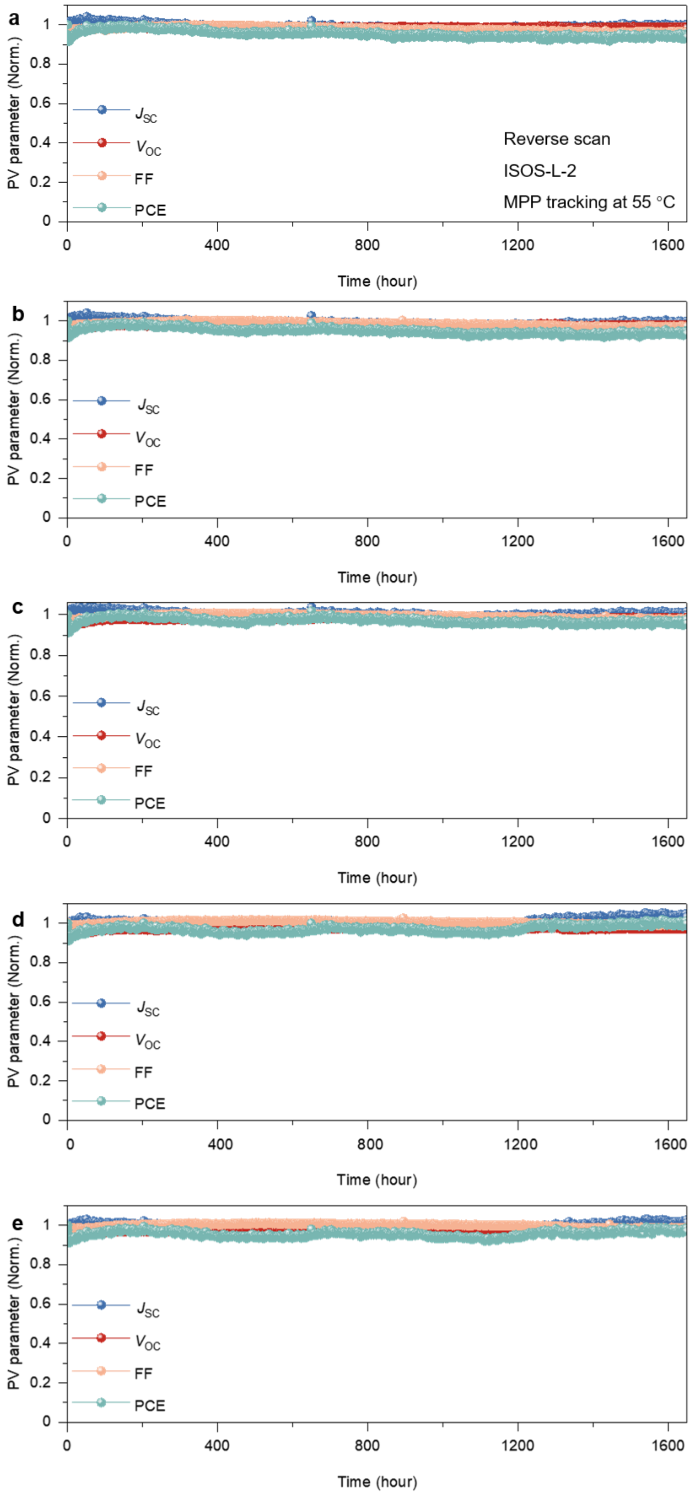

Supplementary Fig. 20 Normalized PV parameters obtained from reverse *J-V* scans over time for devices of ITO/SnO_2/PCBM/perovskite/PDCBT/BCF/Au. **a-e** Normalized PV parameter of (**a**) device 1, (**b**) device 2, (**c**) device 3, (**d**) device 4 and (**e**) device 5. The PSCs were aged at 55 °C in N_2-filled chamber under continuous light illumination (light intensity: 84 mW cm^{-2}) without encapsulation.

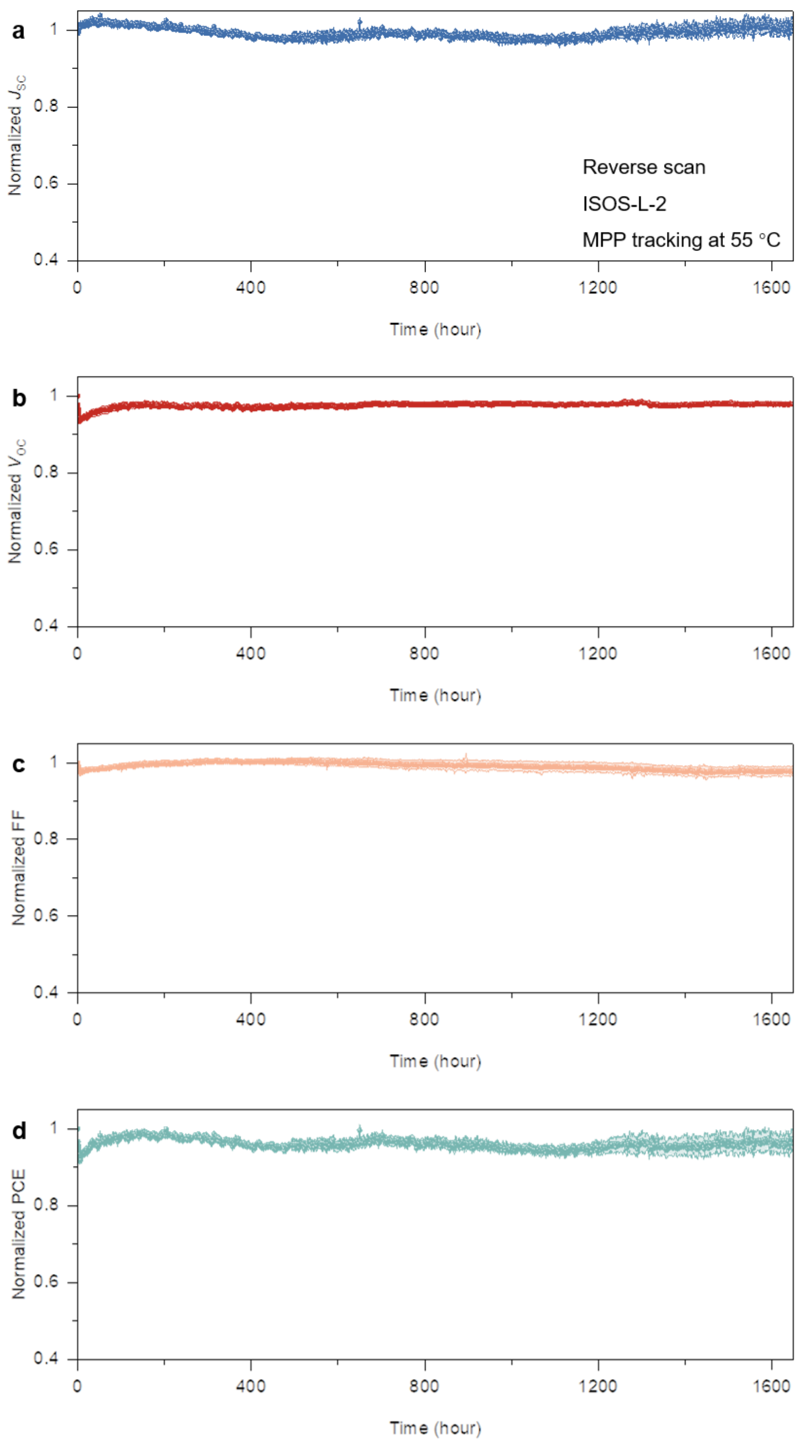

Supplementary Fig. 21 The statistic degradation behavior of PSCs with reverse *J*-*V* scan. **a-d** J_{SC} (**a**), V_{OC} (**b**), FF (**c**) and PCE (**d**) evolutions of PSCs based on ITO/SnO_2/PCBM/perovskite/PDCBT/BCF/Au architecture. The PSCs were aged at 55 °C in N_2-filled chamber under continuous light illumination (light intensity: 84 mW cm^{-2}). Error bars represent the standard deviations from the statistic results of 5 individual devices.

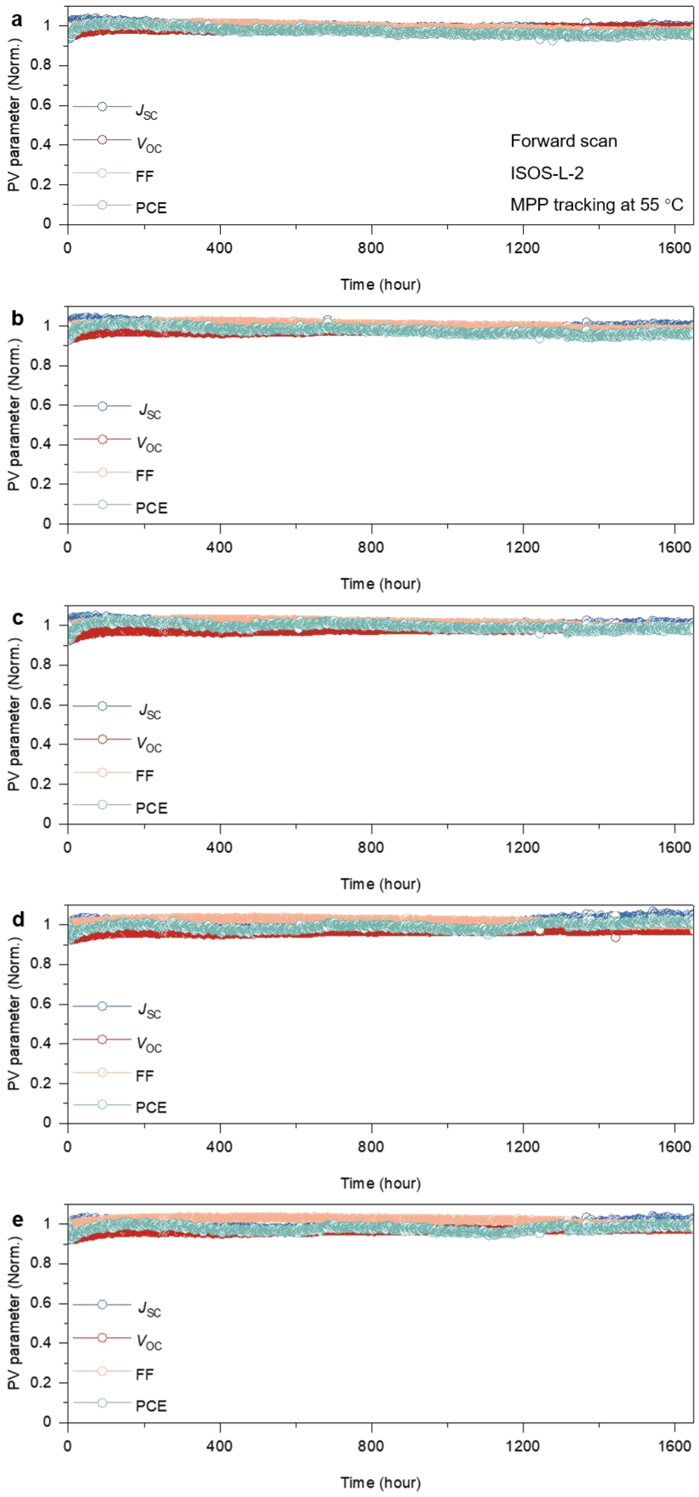

Supplementary Fig. 22 Normalized PV parameters obtained from forward *J-V* scans over time for devices of ITO/SnO_2/PCBM/perovskite/PDCBT/BCF/Au. **a-e** Normalized PV parameter of (**a**) device 1, (**b**) device 2, (**c**) device 3, (**d**) device 4 and (**e**) device 5. The PSCs were aged at 55 °C in N_2-filled chamber under continuous light illumination (light intensity: 84 mW cm^{-2}) without encapsulation.

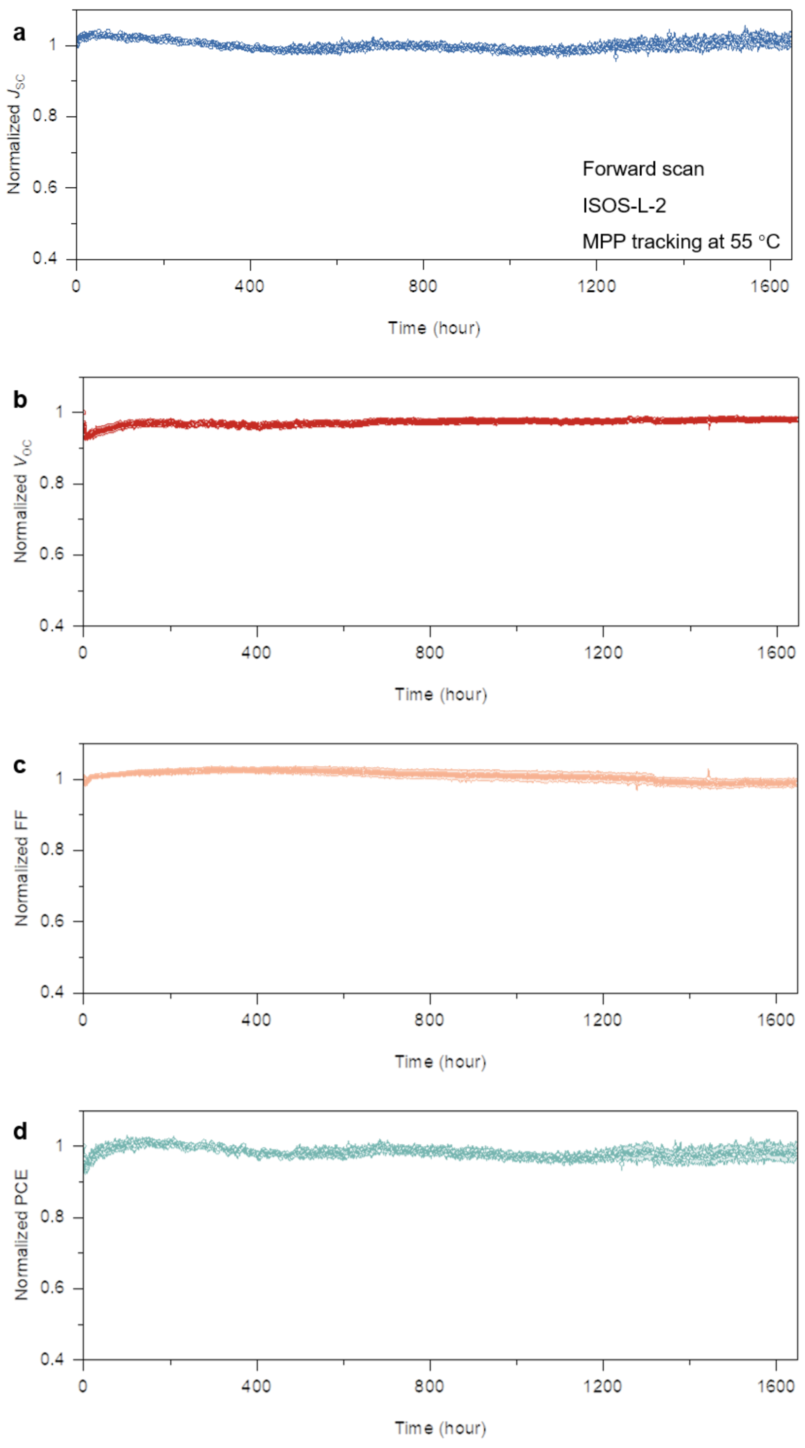

Supplementary Fig. 23 The statistic degradation behavior of PSCs with forward *J-V* scan. **a-d** J_{SC} (**a**), V_{OC} (**b**), FF (**c**) and PCE (**d**) evolutions of PSCs based on ITO/SnO_2/PCBM/perovskite/PDCBT/BCF/Au architecture. The PSCs were aged at 55 °C in N_2-filled chamber under continuous light illumination (light intensity: 84 mW cm^{-2}). Error bars represent the standard deviations from the statistic results of 5 individual devices.

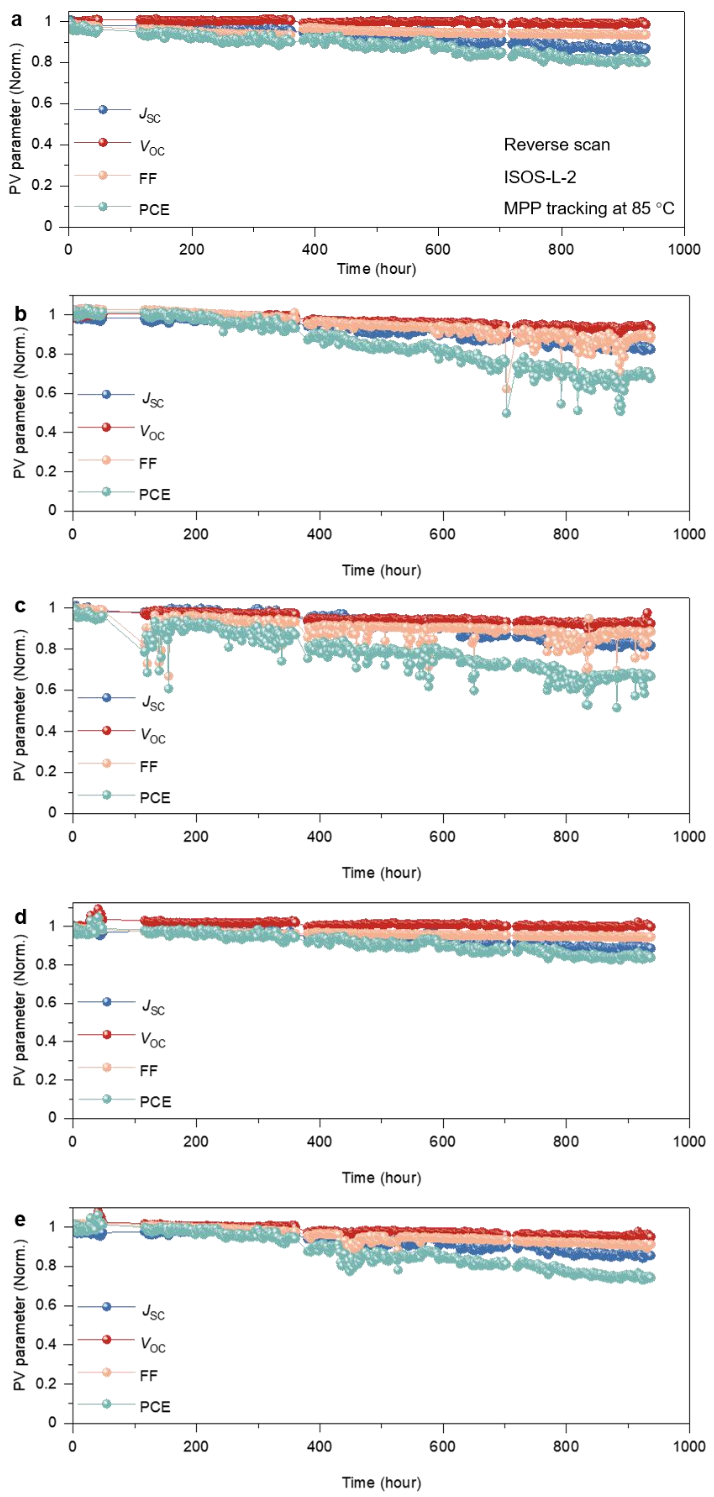

Supplementary Fig. 24 Normalized PV parameters of PSCs based on ITO/SnO_2/PCBM/perovskite/PDCBT/BCF/Au structure. **a-e** Normalized PV parameter of (**a**) device 1, (**b**) device 2, (**c**) device 3, (**d**) device 4 and (**e**) device 5 obtained from reverse *J-V* scans over time. The unencapsulated PSCs were aged at 85 °C in N_2 under continuous light illumination (84 mW cm^{-2}).

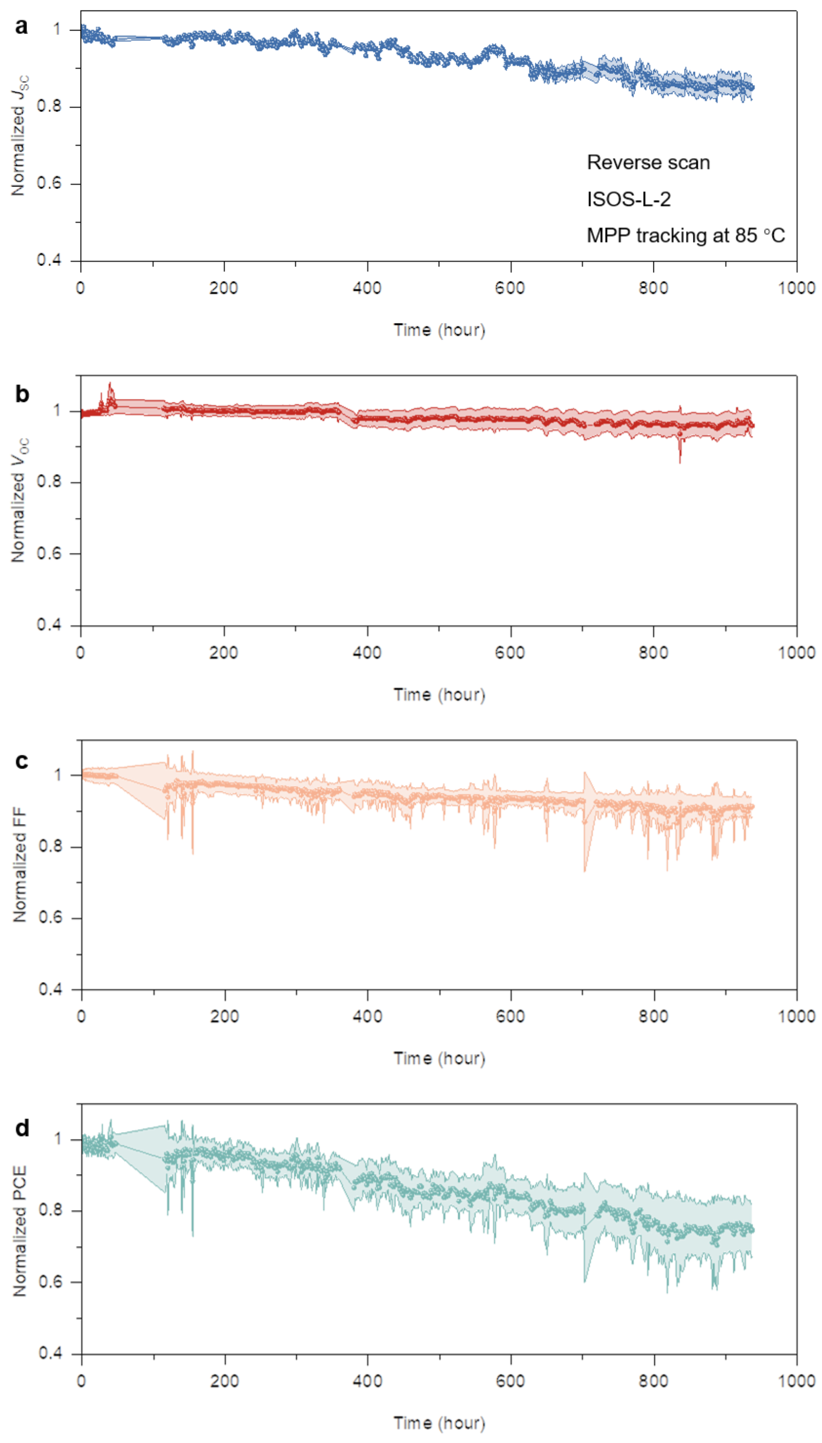

Supplementary Fig. 25 The statistic degradation behavior of PSCs with reverse *J*-*V* scan. **a-d** J_{SC} (**a**), V_{OC} (**b**), FF (**c**) and PCE (**d**) evolutions of PSCs based on ITO/SnO_2/PCBM/perovskite/PDCBT/BCF/Au architecture. The PSCs were aged at 85 °C in N_2-filled chamber under continuous light illumination (light intensity: 84 mW cm^{-2}). Error bars represent the standard deviations from the statistic results of 5 individual devices.

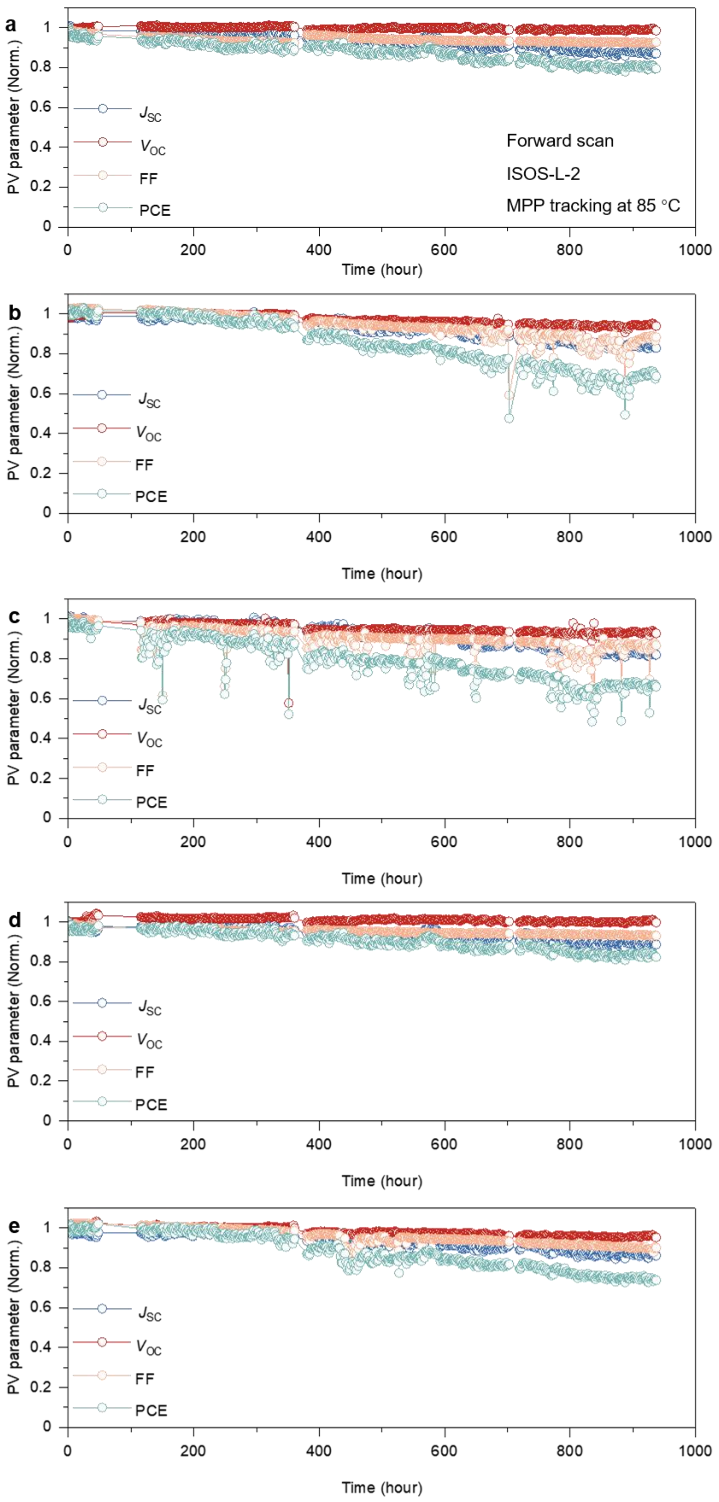

Supplementary Fig. 26 Normalized PV parameters of PSCs based on ITO/SnO_2/PCBM/perovskite/PDCBT/BCF/Au structure. **a-e** Normalized PV parameter of (**a**) device 1, (**b**) device 2, (**c**) device 3, (**d**) device 4 and (**e**) device 5 obtained from forward *J-V* scans over time. The unencapsulated PSCs were aged at 85 °C in N_2 under continuous light illumination (84 mW cm^{-2}).

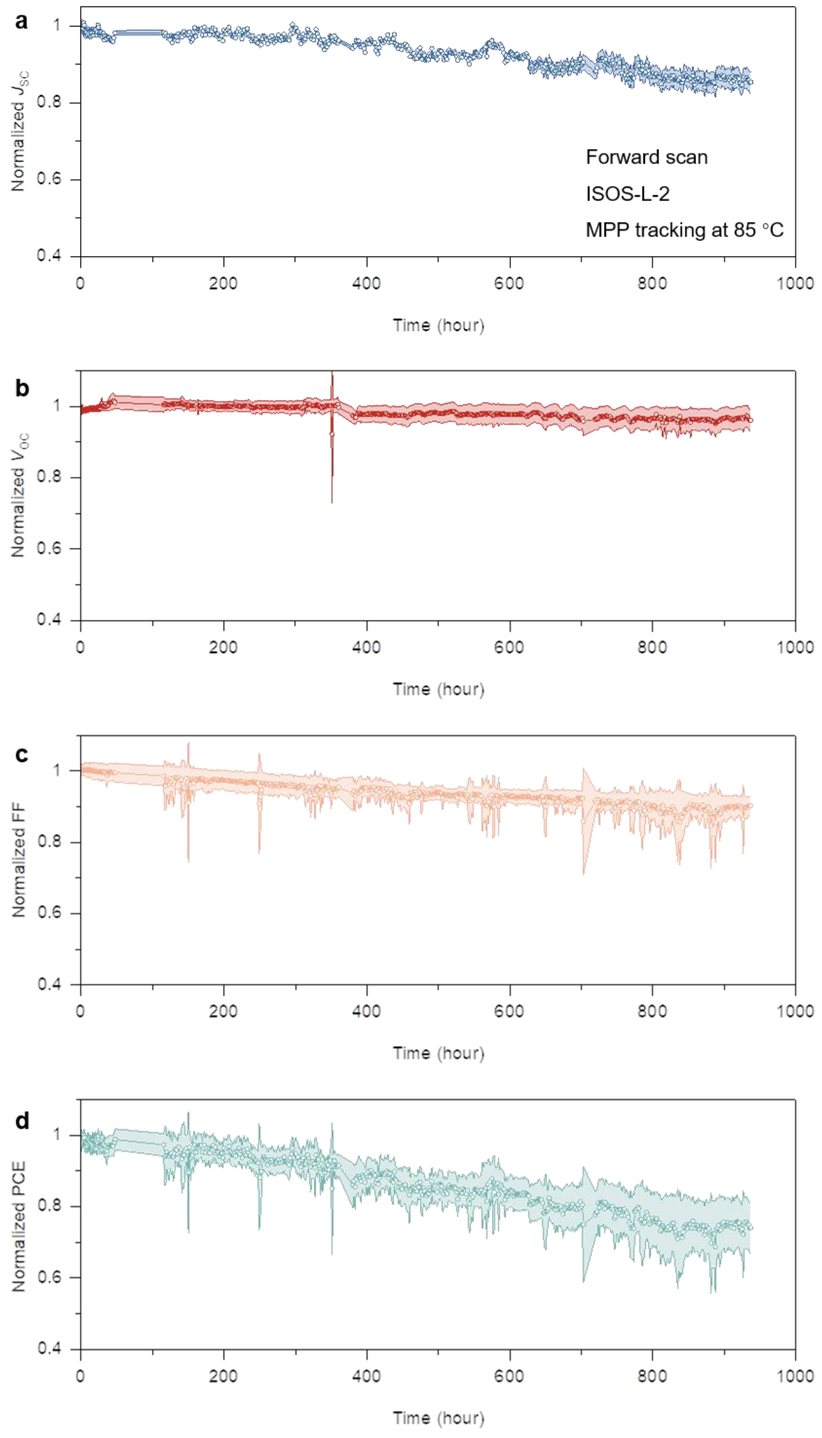

Supplementary Fig. 27 The statistic degradation behavior of PSCs with forward *J-V* scan. **a-d** J_{SC} (**a**), V_{OC} (**b**), FF (**c**) and PCE (**d**) evolutions of PSCs based on ITO/SnO_2/PCBM/perovskite/PDCBT/BCF/Au architecture. The PSCs were aged at 85 °C in N_2-filled chamber under continuous light illumination (light intensity: 84 mW cm^{-2}). Error bars represent the standard deviations from the statistic results of 5 individual devices.

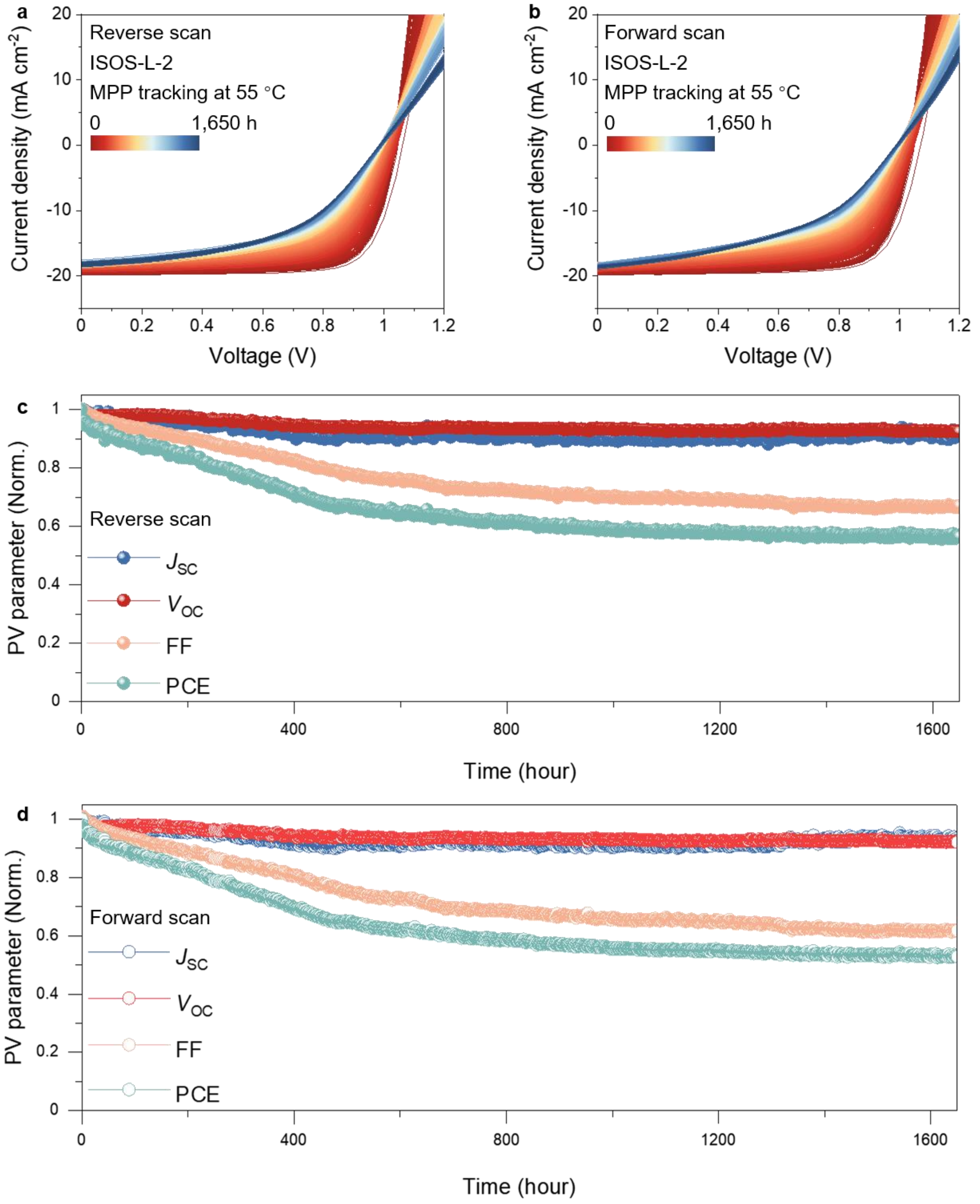

Supplementary Fig. 28 Operational stability of unencapsulated PSCs based on ITO/SnO_2/PCBM/perovskite/PDCBT/Ta-WO_x/Au structure under continuous light illumination (84 mW cm^{-2}) at 55 °C in N_2-filled chamber. **a,b** *J*-*V* curves in (**a**) reverse and (**b**) forward scans for the device from *t* = 0 hour (orange) to *t* = 1,650 hours (Navy). **c,d** Long-term stability of the device in (**c**) reverse and (**d**) forward *J*-*V* scans.

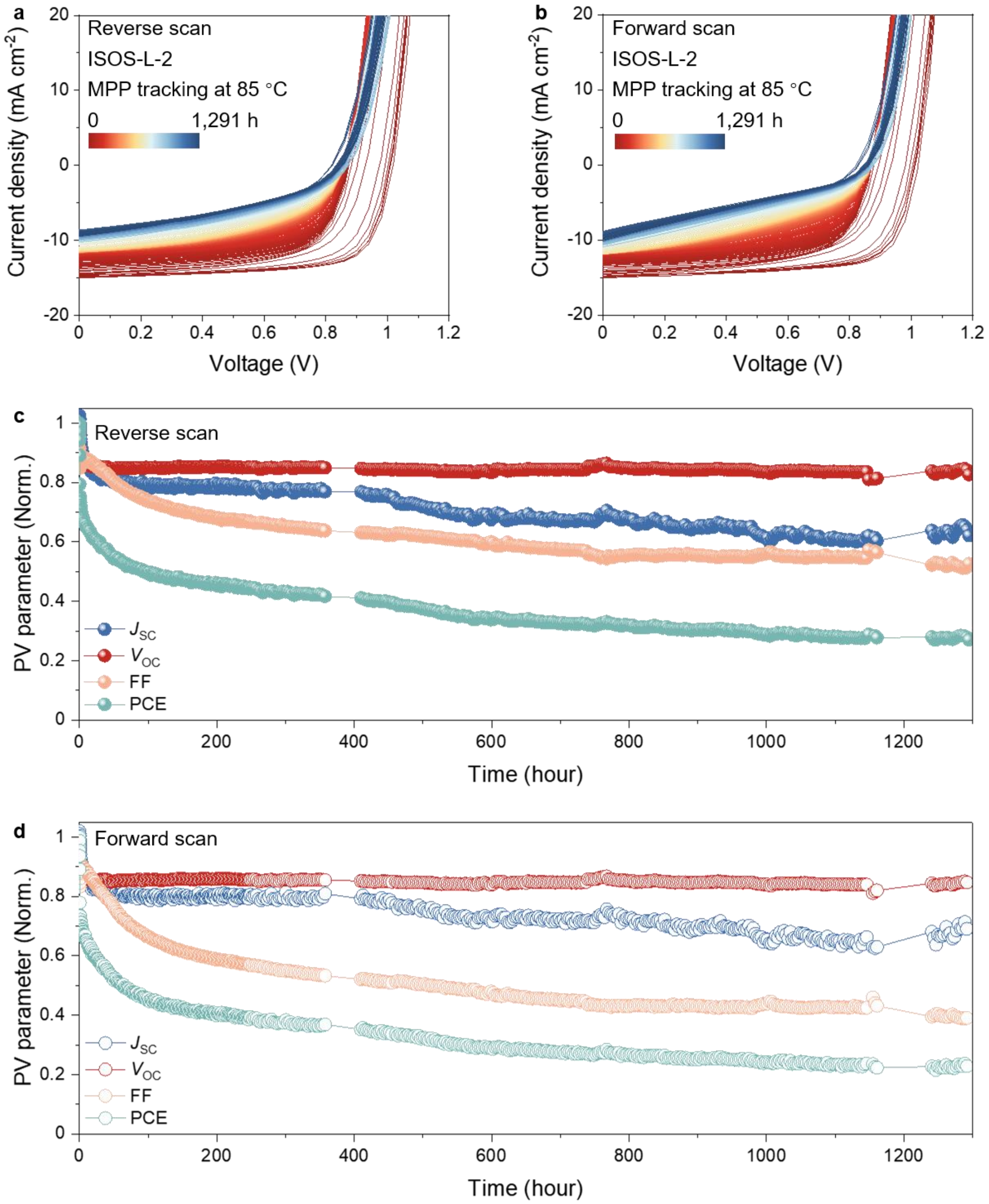

Supplementary Fig. 29 Photo-thermal-operational stability of PSCs based on ITO/SnO_2/PCBM/perovskite/PDCBT/Ta-WO_x/Au structure. **a,b** *J*-*V* curves for the unencapsulated PSC measured with (**a**) reverse and (**b**) forward scans under continuous light illumination (62 mW cm^{-2}) at 85 °C in N_2-filled chamber. **c,d** Photo-thermal-operational stability of the device in (**c**) reverse and (**d**) forward *J*-*V* scans.

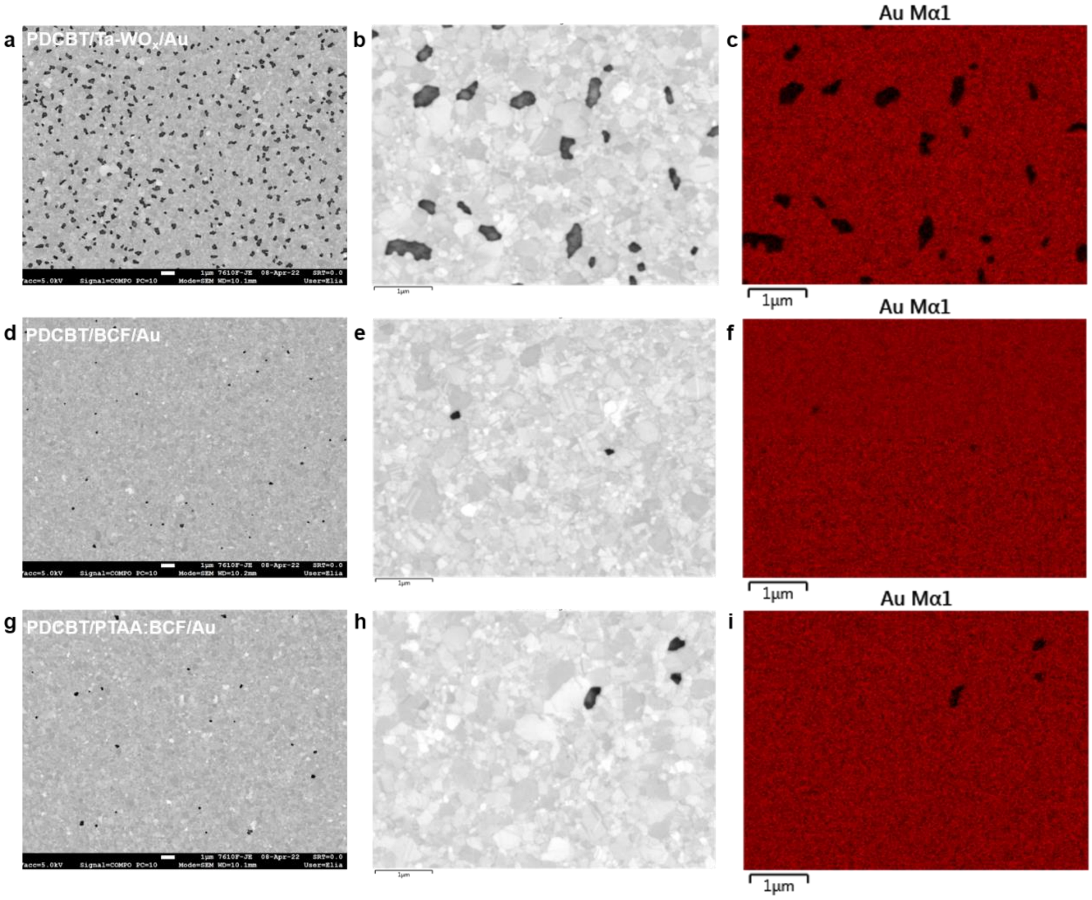

Supplementary Fig. 30 Top-view SEM-BSE images (Z-contrast) and EDX elemental mapping of the Au electrode without MgF_2 capping layer on the aged devices at 85 °C for 10 hours in glovebox. **a-c** The device with structure of ITO/SnO_2/PCBM/perovskite/PDCBT/Ta-WO_x/Au. **d-f** The device with structure of ITO/SnO_2/PCBM/perovskite/PDCBT/BCF/Au. **g-i** The device with structure of ITO/SnO_2/PCBM/perovskite/PDCBT/PTAA:BCF/Au.

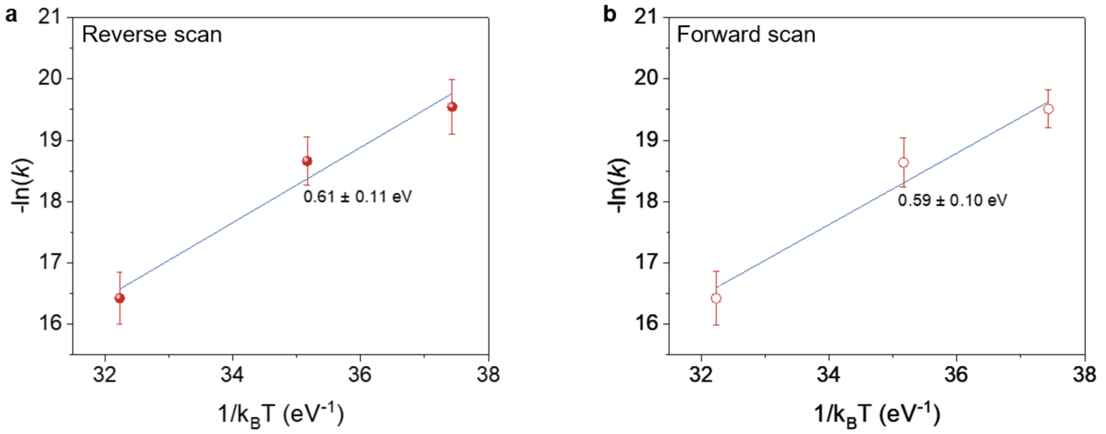

Supplementary Fig. 31 Natural logarithm of degradation rate (*k*) versus $1/k_B T$. **a,b** The data of *k* originated from analyzing *J*-*V* curves with (**a**) reverse and (**b**) forward scans of PSCs based on ITO/SnO_2/PCBM/perovskite/PDCBT/BCF/Au architecture. Error bars represent the standard deviations from the statistic results of 5 individual devices for each temperature.

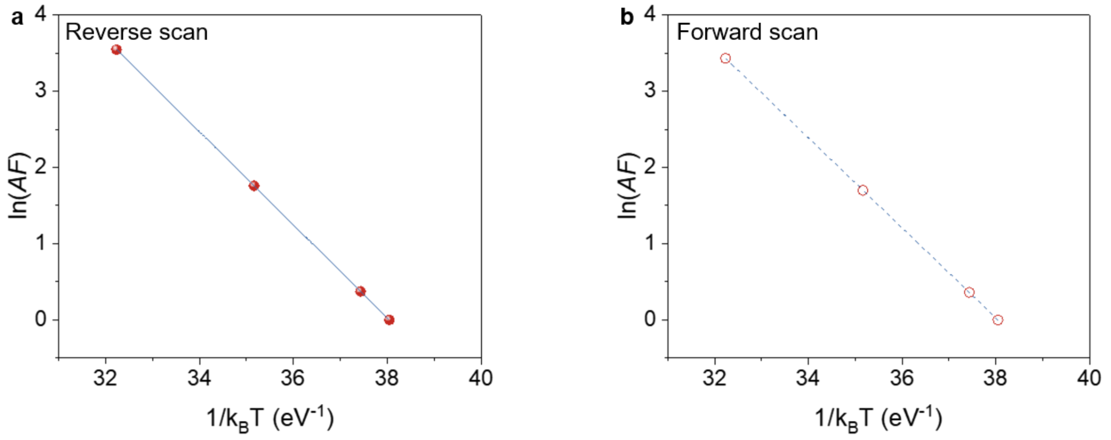

Supplementary Fig. 32 Natural logarithm of *AF* versus $1/k_BT$. **a,b** The value of *AF* was derived from analyzing *J-V* curves with (**a**) reverse and (**b**) forward scans of PSCs based on ITO/SnO_2/PCBM/perovskite/PDCBT/BCF/Au architecture.

Supplementary Table 1 The vendor and price of polymers and Lewis acid BCF.

Product	Vendor	Price ($/g)
PDCBT	1-Material	3000
PDCBT	Solarmer	2500
P3HT	Ossila	350
PTAA	Ossila	1850
BCF	TCI	110

Supplementary Table 2 Comparison of our devices stability with the recently reported stability of PSCs.

Device structure	Aging condition	Efficiency loss	Reference
ITO/SnO_2/PCBM/ $Cs_{0.05}FA_{0.85}MA_{0.1}Pb(I_{0.95}Br_{0.05})_3$/PDCBT/BCF/Au	Encapsulation: No<br>Light intensity: 84 mW cm^{-2}<br>Temperature: 35 °C<br>Test duration: 2,212 hours in N_2	1%	This work
ITO/SnO_2/PCBM/ $Cs_{0.05}FA_{0.85}MA_{0.1}Pb(I_{0.95}Br_{0.05})_3$/PDCBT/BCF/Au	Encapsulation: No<br>Light intensity: 84 mW cm^{-2}<br>Temperature: 55 °C<br>Test duration: 1,650 hours in N_2	2%	This work
ITO/SnO_2/PCBM/ $Cs_{0.05}FA_{0.85}MA_{0.1}Pb(I_{0.95}Br_{0.05})_3$/PDCBT/BCF/Au	Encapsulation: No<br>Light intensity: 84 mW cm^{-2}<br>Temperature: 85 °C<br>Test duration: 937 hours in N_2	18%	This work
ITO/MeO-2PACz/$Rb_{0.05}Cs_{0.05}MA_{0.05}FA_{0.85}Pb(I_{0.95}Br_{0.05})_3$/C60/$SnO_2$/Ag	Encapsulation: No<br>Light intensity: 120 mW cm^{-2}<br>Temperature: 85 °C<br>Test duration: 360 hours in N_2	20%	*Nature* 623, 313-318 (2023)
ITO/PTAA:BCP/$FACsPbI_3$/C60/BCP/Cu	Encapsulation: Yes<br>Light intensity: 100 mW cm^{-2}<br>Temperature: 55-61 °C<br>Test duration: 3,010 hours	10%	*Science* 380, 823-829 (2023)
ITO/SnO_2/KCl/$Cs_{0.05}FA_xMA_{1-x}PbI_3$ /PMMA/Spiro-OMeTAD/Au	Encapsulation: No<br>Light intensity: 100 mW cm^{-2}<br>Temperature: 30-35 °C<br>Test duration:1,000 hours in N_2	10%	*Nature Photonics* 17, 96-105 (2023)

FTO/PTAA/Al_2O_3/$FA_yCs_{1-y}Pb(Br_xI_{1-x})_3$/LiBr/C70/ Zr(acac)$_4$/PEIE/Au	Encapsulation: Yes<br>Light intensity: 76 mW cm^{-2}<br>Temperature: 65 °C<br>Test duration: 1,410 hours	20%	*Nature Materials* 22, 73-83 (2023)
ITO/Me-4PACz/$Cs_{0.05}(FA_{0.98}MA_{0.02})_{0.95}Pb(I_{0.98}Br_{0.02})_3$/LiF/C60/BCP/Ag	Encapsulation: No<br>Light intensity: 100 mW cm^{-2}<br>Temperature: 40-45 °C<br>Test duration: 1,200 hours in N_2	9%	*Nature Energy* 8, 462-472 (2023)
FTO/SnO_2/$FAPbI_3$/Spiro-OMeTAD/Au	Encapsulation: Yes<br>Light intensity: 100 mW cm^{-2}<br>Temperature: 25 °C<br>Test duration: 600 hours in N_2	12%	*Nature* 616, 724-730 (2023)
FTO/SnO_2/$FAPbI_3$/CBz-PAI/Spiro-OMeTAD/Au	Encapsulation: No<br>Light intensity: 100 mW cm^{-2}<br>Temperature: 45 °C<br>Test duration: 1,100 hours in N_2	5%	*Nature Energy* 8, 515-525 (2023)
ITO/MeO-2PACz)/ $Cs_{0.05}(FA_{0.98}MA_{0.02})_{0.95}Pb(I_{0.98}Br_{0.02})_3$ /PC61BM/BCP/Ag	Encapsulation: No<br>Light intensity: 100 mW cm^{-2}<br>Temperature: 25 °C<br>Test duration: 1,000 hours in N_2	4%	*Science* 379, 399-403 (2023)
ITO/NiO_x/$Cs_{0.05}FA_{0.85}MA_{0.1}PbI_3$/PCBM/BCP/Ag	Encapsulation: Yes<br>Light intensity: 100 mW cm^{-2}<br>Temperature: 65 °C<br>Test duration: 500 hours	8%	*Nature Photonics* 16, 352-358 (2022)
ITO/SnO_2/$Cs_{0.1}FA_{0.9}PbI_3$/Spiro-OMeTAD/Au	Encapsulation: No<br>Light intensity: 100 mW cm^{-2}<br>Temperature: 45 ± 5 °C<br>Test duration: 3,190 hours in N_2	9%	*Science* 378, 747-754 (2022)
FTO/TiO_2/$FA_{0.85}MA_{0.1}Cs_{0.05}PbI_{2.9}Br_{0.1}$/Spiro-OMeTAD/Au	Encapsulation: No<br>Light intensity: 100 mW cm^{-2}<br>Temperature: 40 °C<br>Test duration: 1,000 hours in N_2	10%	*Science* 375, 71-76 (2022)
