# Supplementary material for: Polymer-acid-metal quasi-ohmic contact for stable perovskite solar cells beyond a 20,000-hour extrapolated lifetime
Source: Nat Commun. 2024 Mar 5;15:2002. doi: 10.1038/s41467-024-46145-7 (PMC10914746; doi:10.1038/s41467-024-46145-7)
Supplement: Supplementary file 3 — Reporting Summary [file 41467_2024_46145_MOESM3_ESM.pdf]

nature portfolio

Corresponding author(s): Zhongquan Wan, Chunyang Jia, Christoph J. Brabec and Yicheng Zhao

Solar Cells Reporting Summary

Nature Portfolio wishes to improve the reproducibility of the work that we publish. This form is intended for publication with all accepted papers reporting the characterization of photovoltaic devices and provides structure for consistency and transparency in reporting. Some list items might not apply to an individual manuscript, but all fields must be completed for clarity.

For further information on Nature Research policies, including our data availability policy, see Authors & Referees.

Experimental design

Please check the following details are reported in the manuscript, and provide a brief description or explanation where applicable.

1. Dimensions

Area of the tested solar cells

☒ Yes — The designed device area is 0.12 cm^2 and is tested with an aperture of 0.113 cm^2.
☐ No — *Explain why this information is not reported/not relevant.*

Method used to determine the device area

☒ Yes — Method: The area of aperture mask is 0.113 cm^2.
☐ No — *Explain why this information is not reported/not relevant.*

2. Current-voltage characterization

Current density-voltage (J-V) plots in both forward and backward direction

☒ Yes — Method: Negligible hysteresis is observed for all the measurements.
☐ No

Voltage scan conditions

☒ Yes — Method: The J-V characteristics were measured from 0 to 1.2 V (forward scan) and 1.2 to 0 V (reverse scan) at a scan rate of 20 mV/s.
☐ No — *Explain why this information is not reported/not relevant.*

Test environment

☒ Yes — Method: The devices were tested in air at room temperature.
☐ No — *Explain why this information is not reported/not relevant.*

Protocol for preconditioning of the device before its characterization

☐ Yes — *Provide a description of the protocol.*
☒ No — There is no light soaking effect and device shows negligible hysteresis.

Stability of the J-V characteristic

☒ Yes — Method: For maximum power point (MPP) tracking, the MPP tracking point is based on the reverse J-V scan from 1.2 to 0 V every 1-3 hours. Both forward and reverse scan are recorded.
☐ No — *Explain why this information is not reported/not relevant.*

3. Hysteresis or any other unusual behaviour

Description of the unusual behaviour observed during the characterization

☐ Yes — *Provide a description of hysteresis or any other unusual behaviour observed during the characterization.*
☒ No — Negligible hysteresis is observed in our devices.

Related experimental data

☒ Yes — The data are presented in Figs. 3 and 4 and Supplementary Figs. 12, 13, 28 and 29.
☐ No — *Explain why this information is not reported/not relevant.*

4. Efficiency

External quantum efficiency (EQE) or incident photons to current efficiency (IPCE)

☒ Yes — The data are presented in Fig. 3 in the main text.
☐ No — *Explain why this information is not reported/not relevant.*

A comparison between the integrated response under the standard reference spectrum and the response measure under the simulator

☒ Yes — The data are presented in Fig. 3 in the main text.
☐ No — *Explain why this information is not reported/not relevant.*

For tandem solar cells, the bias illumination and bias voltage used for each subcell

☐ Yes — *Provide a description of the measurement conditions.*
☒ No — Not applicable.

5. Calibration

Light source and reference cell or sensor used for the characterization

☒ Yes — Method.
☐ No — *Explain why this information is not reported/not relevant.*

Confirmation that the reference cell was calibrated and certified

☒ Yes — Method.
☐ No — *Explain why this information is not reported/not relevant.*

Calculation of spectral mismatch between the reference cell and the devices under test

☒ Yes — Spectral mismatch factor of 1 is used for all the J-V measurements.
☐ No — *Explain why this information is not reported/not relevant.*

6. Mask/aperture

Size of the mask/aperture used during testing

☒ Yes — Method: The area of aperture mask is 0.113 cm^2.
☐ No — *Explain why this information is not reported/not relevant.*

Variation of the measured short-circuit current density with the mask/aperture area

☐ Yes — *Report the difference in the short-circuit current density values measured with the mask and aperture area.*
☒ No — Not applicable.

7. Performance certification

Identity of the independent certification laboratory that confirmed the photovoltaic performance

☐ Yes — *Identify the independent certification laboratory.*
☒ No — 1. The light source of simulated AM1.5G is calibrated by a certified silicon solar cell.
2. The devices are almost hysteresis-free.
3. The focus of this work is the operational stability of solar cells.

A copy of any certificate(s)

☐ Yes — *Certificate copies should be provided in the Supplementary information. Please state the supplementary item number.*
☒ No — Not applicable.

8. Statistics

Number of solar cells tested

☒ Yes — 20 devices, Fig. 3 and Supplementary Fig. 14.
☐ No — *Explain why this information is not reported/not relevant.*

Statistical analysis of the device performance

☒ Yes — Fig. 3 and Supplementary Fig. 14.
☐ No — *Explain why this information is not reported/not relevant.*

9. Long-term stability analysis

Type of analysis, bias conditions and environmental conditions

☒ Yes — Following the International Summit on Organic Photovoltaic Stability protocols, the stability of our devices is measured at the MPP with different temperatures (35, 55 and 85 °C) under continuous light illumination in home-made chambers with flowing N2.
☐ No — *Explain why this information is not reported/not relevant.*
